# Comparative Metagenomics of Cellulose- and Poplar Hydrolysate-Degrading Microcosms from Gut Microflora of the Canadian Beaver (*Castor canadensis*) and North American Moose (*Alces americanus*) after Long-Term Enrichment

**DOI:** 10.3389/fmicb.2017.02504

**Published:** 2017-12-20

**Authors:** Mabel T. Wong, Weijun Wang, Marie Couturier, Fakhria M. Razeq, Vincent Lombard, Pascal Lapebie, Elizabeth A. Edwards, Nicolas Terrapon, Bernard Henrissat, Emma R. Master

**Affiliations:** ^1^Department of Chemical Engineering and Applied Chemistry, University of Toronto, Toronto, ON, Canada; ^2^Centre de Recherches sur les Macromolécules Végétales – Université Grenoble Alpes, Grenoble, France; ^3^Centre National de la Recherche Scientifique, Centre de Recherches sur les Macromolécules Végétales, Grenoble, France; ^4^Architecture et Fonction des Macromolécules Biologiques, Aix-Marseille Université, Marseille, France; ^5^UMR 7257, Centre National de la Recherche Scientifique, Marseille, France; ^6^Department of Biological Sciences, King Abdulaziz University, Jeddah, Saudi Arabia; ^7^Department of Bioproducts and Biosystems, Aalto University, Espoo, Finland

**Keywords:** comparative metagenomics, lignocellulose degradation, carbohydrate-active enzymes (CAZymes), polysaccharide utilization loci (PULs), microbial enrichment, digestive microbiome, beaver, moose

## Abstract

To identify carbohydrate-active enzymes (CAZymes) that might be particularly relevant for wood fiber processing, we performed a comparative metagenomic analysis of digestive systems from Canadian beaver (*Castor canadensis*) and North American moose (*Alces americanus*) following 3 years of enrichment on either microcrystalline cellulose or poplar hydrolysate. In total, 9,386 genes encoding CAZymes and carbohydrate-binding modules (CBMs) were identified, with up to half predicted to originate from *Firmicutes, Bacteroidetes, Chloroflexi*, and *Proteobacteria* phyla, and up to 17% from unknown phyla. Both PCA and hierarchical cluster analysis distinguished the annotated glycoside hydrolase (GH) distributions identified herein, from those previously reported for grass-feeding mammals and herbivorous foragers. The CAZyme profile of moose rumen enrichments also differed from a recently reported moose rumen metagenome, most notably by the absence of GH13-appended dockerins. Consistent with substrate-driven convergence, CAZyme profiles from both poplar hydrolysate-fed cultures differed from cellulose-fed cultures, most notably by increased numbers of unique sequences belonging to families GH3, GH5, GH43, GH53, and CE1. Moreover, pairwise comparisons of moose rumen enrichments further revealed higher counts of GH127 and CE15 families in cultures fed with poplar hydrolysate. To expand our scope to lesser known carbohydrate-active proteins, we identified and compared multi-domain proteins comprising both a CBM and domain of unknown function (DUF) as well as proteins with unknown function within the 416 predicted polysaccharide utilization loci (PULs). Interestingly, DUF362, identified in iron–sulfur proteins, was consistently appended to CBM9; on the other hand, proteins with unknown function from PULs shared little identity unless from identical PULs. Overall, this study sheds new light on the lignocellulose degrading capabilities of microbes originating from digestive systems of mammals known for fiber-rich diets, and highlights the value of enrichment to select new CAZymes from metagenome sequences for future biochemical characterization.

## Introduction

Lignocellulose comprises the non-edible fraction of plant biomass and as such is a recognized resource for the production of renewable energy, chemicals, and materials. The main components of lignocellulose are cellulose, hemicellulose, pectin, and lignin, the proportions and particular chemistries of which depend largely on the plant type and fraction (Kumar et al., 2008). For instance, glucuronoarabinoxylan is a typical hemicellulose found in agricultural crops, whereas glucuronoxylan is the predominant hemicellulose in wood tissue of deciduous trees, including poplar (Peng et al., 2012). Bioconversion pathways that convert various lignocellulose sources to targeted end products require the concerted action of CAZymes, which include glycosyl hydrolases (GHs), carbohydrate esterases (CEs), polysaccharide lyases (PLs), auxiliary activities (AAs), and of carbohydrate binding modules (CBMs) that are classified into families according to amino acid sequence similarity in the CAZy database^1^ (Lombard et al., 2014). Various pretreatment methods, including steam explosion, have been developed to maximize enzymatic conversion of lignocellulosic resources (Excoffier et al., 1991).

Metagenomic approaches to identify CAZymes relevant to the conversion of a given biomass feedstock have considered environmental samples persistently subjected to the targeted feedstock. For example, metagenomic analyses aimed at identifying CAZymes most relevant to bioconversion of non-woody biomass have sampled digestive systems of animals that graze on straw, grasses, and lichens (Pope et al., 2010). Parallel metagenomic analyses to identify CAZymes contributing to bioconversion of woody biomass have included samples ranging from forest soils (Damon et al., 2012; Pold et al., 2016), to insects (Warnecke et al., 2007; He et al., 2013; Rossmassler et al., 2015), and wood-feeding mollusks (O’Connor et al., 2014). Alternatively, enrichment of environmental samples on specific biomass feedstocks prior to metagenome sequencing can improve sequence assembly (DeAngelis et al., 2010), while facilitating the identification of most pertinent CAZymes (van der Lelie et al., 2012). In a few cases, direct comparison of CAZyme profiles has also been performed for enrichment cultures originating from the same source. Examples include soil-derived microbial communities enriched with wheat straw, switchgrass, and corn stover (Jimenéz et al., 2016), and those digesting mixed lignocellulosic substrates in stationary versus submerged and agitated conditions (Heiss-Blanquet et al., 2016; Wang et al., 2016). In addition to the influence of enrichment condition, such metagenomic studies highlight the increase in number of CAZyme sequences from families associated with hydrolysis of oligosaccharides and side groups of hemicelluloses and/or pectins (e.g., GH3, GH43).

Aside from identifying CAZyme families most pertinent to conversion of specific biomass feedstocks, metagenomic analysis of biomass-degrading communities has uncovered a diverse array of encoded polysaccharide utilization loci (PULs) and multi-modular proteins. Briefly, PULs comprise physically linked genes that encode CAZymes and other proteins that work in concert to degrade specific glycans. Accordingly, PULs have emerged as especially fruitful regions within metagenome sequences for enzyme discovery (Larsbrink et al., 2016; Patrascu et al., 2017). For instance, in the past year alone, detailed biochemical characterization of PULs with different selectivity has uncovered novel activities that contribute to the degradation of pectin (Ndeh et al., 2017), xylan (Wang et al., 2016), and galactomannan (Bagenholm et al., 2017), as well as fungal cell wall components including chitin (Larsbrink et al., 2016) and β-glucans (Temple et al., 2017). Likewise, multi-modular proteins and cellulosomal subunits identified from metagenome sequences and bacterial isolates constitute an additional reservoir for CAZyme discovery (Zhang et al., 2014). Most recently, CAZyme-linked dockerins were reported in PULs in a moose rumen microbiome (Svartström et al., 2017).

With the aim to identify CAZymes and novel proteins that target woody biomass, here we applied a comparative metagenomics approach to identify microbial enzymes encoded by the gut digestive microflora of wood-feeding Canadian beaver (*Castor canadensis*) and North American moose (*Alces americanus*) that are likely to promote the conversion of pretreated wood chips. Briefly, in winter months especially, beavers apply an obligate woody diet consisting of twigs, bark, and tree trunks; such seasonal confinement to a wood-based diet is also common to moose, ungulates that consume twigs, shrubs, and bark during the winter (Chaney, 2003; Hood and Bayley, 2009). In an earlier study, we confirmed the existence of biomass-degrading microorganisms in gut digestive microflora of beaver and moose, and their ability to transform lignocellulosic substrates under anaerobic conditions (Wong et al., 2016). Herein, we report the metagenomes of corresponding gut digestive microflora enriched for over 3 years on either microcrystalline cellulose or pretreated poplar wood chips. In particular, metagenome sequences were compared to reveal CAZyme families that are consistently enriched following growth on poplar hydrolysate compared to growth on cellulose. The four metagenomes were also mined in an effort to identify novel candidate enzymes for future characterization. Two approaches were devised to facilitate this analysis: (1) prediction of bacterial PULs and analysis of encoded proteins with unknown function within PULs, and (2) prediction of multi-modular proteins that comprise both modules recognized to contribute to polysaccharide conversion (e.g., a carbohydrate-binding domain) and domains with unknown function.

## Materials and Methods

### Ethics Statement

An ethics approval from an Animal Care and Use Committee was not required by the Office of Research Ethics of the University of Toronto, as the moose rumen sample was collected from a dead moose that was hunted in the wild for meat by a registered hunter with a license authorized by the Ministry of Natural Resources and Forestry under Government of Ontario, Canada.

### Setup and Maintenance of Lignocellulose Active Enrichment Cultures

As previously described (Wong et al., 2016), lignocellulose-degrading microorganisms from the digestive systems of Canadian beaver (*Castor canadensis*) and North American moose (*Alces americanus*) were sampled and enriched under anaerobic conditions at 36°C. Briefly, approximately 15 ml of the beaver dropping and moose rumen inocula were transferred to separate 160 mM Wheaton glass serum bottles, which were amended with 45 mL of sulfide-reduced mineral medium and 36 mg COD equivalents of microcrystalline cellulose (Avicel PH101; purchased from Sigma–Aldrich) or steam exploded poplar hydrolysate (provided by SunOpta Inc., Canada) (Wong et al., 2016). Biogas production by resulting cultures was carefully monitored to track metabolic activity.

### Metagenomic DNA Extraction and Sequencing

Following 3 years of cultivation and 10 enrichment phases, 10 ml of each enrichment culture was harvested at early stationary phase of biogas production. Samples were centrifuged at 15,000 × *g* for 15 min at 4°C, and total community DNA was extracted using the QIAamp DNA Stool Mini Kit (Qiagen, Hilden, Germany) (Supplementary Table S1). The concentration and quality of the extracted metagenomic DNA were assessed by measuring the 260/280 absorbance ratio using a Nanodrop 2000 Spectrophotometer (Thermo Scientific, MA, United States), and then stored at -80°C. A TruSeq library was constructed for each DNA sample. Illumina paired-end sequencing was chosen and performed with Illumina HiSeq 2000 (Illumina Inc., San Diego, CA, United States) at Génome Québec Innovation Centre.

### Metagenome Assembly

The output reads were processed by Trimmomatic 0.32 for the removal of adapters and quality filtering (Bolger et al., 2014). The quality-trimmed metagenomic reads were assembled using ABySS with minimum coverage of 20, and minimum kmer length of 96 nucleotides (Simpson et al., 2009). Using an in house Perl script, NKD of the assembled contigs were calculated using the formula NKD = *n*/(*L*-*k*+1), where *n* is the total number of kmers assembled to the contig, *L* is the contig length, and *k* is the kmer length (96) used in the assembly. Contig distributions were then visualized by plotting the calculated NKD against contig length.

### Annotation of CAZyme Families, Multi-Modular Sequences, and Polysaccharide Utilization Loci (PULs)

Assembled contigs were subjected to ORF prediction using Prodigal (Hyatt et al., 2010); predicted proteins were then assigned to CAZyme families using a combination of BLAST and HMM searches against CAZy reference sequences and families as already described (Al-Masaudi et al., 2017). Counts of CAZyme sequences were normalized to compare the diversity of CAZyme sequences identified within each enrichment culture. Specifically, since the number of predicted ORFs was highest for the metagenome of beaver droppings enriched on poplar hydrolysate (BD-PH, **Table 1**) then:

**Table 1:** Statistics of the sequencing and assembly of the metagenomes of cellulose (C) and poplar hydrolysate (PH)-fed enrichment cultures from beaver dropping (BD) and moose rumen (MR).

Inocula	Beaver droppings		Moose rumen	
Enrichment	Cellulose	Poplar hydrolysate	Cellulose	Poplar hydrolysate
Abbreviation	BD-C	BD-PH	MR-C	MR-PH
Number of quality trimmed reads (% passed)	74,999,337 (99.7%)	78,144,385 (99.7%)	71,980,296 (99.8%)	88,305,224 (99.8%)
Total Mbp assembled (Mbp)	78	81.5	58.3	67.6
Number of contigs	5,010	10,553	5,705	6,941
Longest contig (bp)	1,266,318	674,797	1,299,156	1,131,879
N50 (bp)	92,758	68,167	71,246	106,046
Number of ORFs	71,348	81,969	56,127	66,970
Normalized counts^1^ of plant polysaccharide-active				
CAZymes (% of all CAZymes)	709 (23.4%)	830 (32.3%)	525 (22.4%)	834 (32.7%)
PUL	38	171	47	42
Type 1 dockerin	95	116	68	94
Type 1 cohesin	42	50	35	41

*^1^Counts were normalized by the number of ORFs.*

$$\mathrm{Normalized}\;\mathrm{count}\;=\;\frac{\begin{array}{c} \mathrm{count}\;\mathrm{of}\;\mathrm{identified}\;\mathrm{sequences}\;\mathrm{in}\;a\;\mathrm{given}\;\mathrm{CAZyme}\;\mathrm{family}\;\\ \mathrm{within}\;a\;\mathrm{metagenome}\;\mathrm{of}\;\mathrm{interest}\;\times \;\mathrm{number}\;\mathrm{of}\;\mathrm{ORFs}\;\\ \mathrm{of}\;BD-PH\;\mathrm{metagenome} \end{array}}{\begin{array}{c} \mathrm{number}\;\mathrm{of}\;\mathrm{ORFs}\;\mathrm{of}\;a\;\mathrm{metagenome}\;\mathrm{of}\;\mathrm{interest} \end{array}}$$

Relative abundance for a given CAZyme family in a metagenome was calculated by

$$\mathrm{Relative}\;\mathrm{abundance}\;=\;\frac{\begin{array}{c} \mathrm{identified}\;\mathrm{counts}\;\mathrm{for}\;a\;\mathrm{given}\;\mathrm{CAZyme}\;\mathrm{family}\;\mathrm{in}\;a\;\\ \mathrm{metagenome} \end{array}}{\begin{array}{c} \mathrm{total}\;\mathrm{identified}\;\mathrm{counts}\;\mathrm{of}\;\mathrm{CAZyme}\;\mathrm{in}\;a\;\\ \mathrm{metagenome} \end{array}}\times 100\%$$

Public metagenomes from the digestive systems of cow (Hess et al., 2011), moose (Svartström et al., 2017), panda (Zhu et al., 2011), reindeer (Pope et al., 2012), Saudi sheep (Al-Masaudi et al., 2017), termite (Warnecke et al., 2007), and wallaby (Pope et al., 2010) were also reannotated based on the latest version of the CAZy database and included in this calculation. Relative abundances of predicted plant (poly)saccharide-active CAZyme families (**Figure 4**) were then extracted for hierarchical clustering (correlation clustering and average linkage) and PCA using R statistics in ClustVis (Metsalu and Vilo, 2015). Taxonomic assignment of predicted CAZymes from each metagenome was determined using protein sequences belonging to archaea, bacteria, fungi, other microbial eukaryotes, and viruses reported in the NCBI-NR database (downloaded 16 May 2017) using Kaiju in greedy mode with default settings (Menzel et al., 2016). The phylogenetic distributions of the top 10 identified organisms were visualized at phylum and class levels in a chord diagram using Circos (Krzywinski et al., 2009).

Cellulosomal modules (dockerin and cohesin domains) as well as S-layer homology domains were identified using reference sequences and models built from the literature (Mesnage et al., 2000; Venditto et al., 2016; Artzi et al., 2017). Proteins with CBMs appended to >200 amino acids not covered by any CAZy module were subjected to Pfam domain annotation (Finn et al., 2016) using InterProScan (Jones et al., 2014) to identify conserved DUF (Goodacre et al., 2014). PULs were predicted around *susCD*-like genes, and boundaries were extended based on intergenic distances, the presence of CAZymes and of regulatory genes (e.g., hybrid two-component system protein, extracytoplasmic function (ECF) σ/anti-σ factors, etc.) following the automatic method used in PULDB (Terrapon et al., 2015). The proteins with unknown functions from the PULs reported here and in PULDB^2^ were pooled and submitted to CD-HIT web server (Huang et al., 2010) to identify proteins that meet a similarity threshold, which was defined by being ≥70% identical to the representative sequence, and having ≥70% alignment coverage. The sequence reads were submitted to NCBI under the Bioproject ID SUB1022597.

## Results and Discussion

### Metagenomic DNA Extraction and Sequencing Statistics

Each metagenomic DNA sample (Supplementary Table S1) yielded 71–88 Mb high-quality reads (150 bp long each), which were assembled into 5,010 to 10,553 contigs per metagenome (**Table 1**). The N50 (i.e., the length at which 50% of the assembled contigs were equal to or longer than) ranged between 68 and 105 kbp, and longest contigs were between 674 kbp and 1.13 Mbp. As shown in the summary of contig profiles (Supplementary Figure S1), longer contigs were generally present at lower NKD.

### Comparison of Predicted CAZyme Sequences with Existing Data Sets

In total, 9,386 genes encoding CAZymes and CBMs were predicted from the four metagenomes (Supplementary Table S2). These sequences were assigned to 100 distinct families of GHs, 13 families of CEs, 15 families of PLs, and 39 families of GTs, as well as 43 families of associated CBMs. As observed for other anaerobic microbial communities (Al-Masaudi et al., 2017; Svartström et al., 2017), no auxiliary redox enzymes were identified.

**FIGURE 1:**
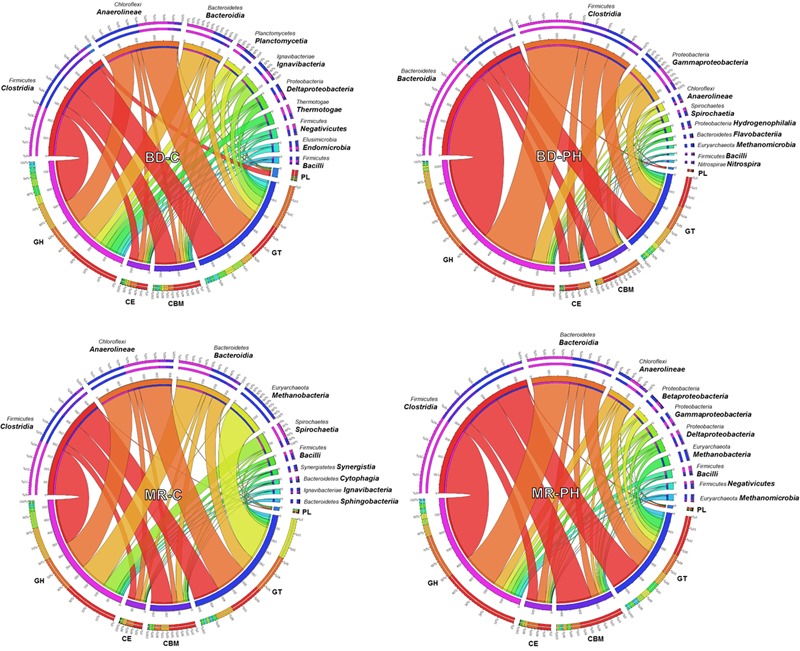
Phylogenetic distribution of CAZyme sequences assigned to the top 10 identified classes.

On average, 13% (up to 17% in cellulose-fed moose rumen culture) of the annotated CAZymes were taxonomically unassigned or assigned to unknown species. The phylogenetic origins of the remaining varied among the metagenomes, with most of them derived from *Firmicutes, Bacteroidetes, Chloroflexi*, and *Proteobacteria* phyla (**Figure 1**). Up to 14% of predicted CAZyme sequences from cellulose-fed enrichments were assigned to class *Anaerolineae*, whereas CAZyme assignments to class *Gammaproteobacteria* were unique to cultures fed on poplar hydrolysate (**Figure 1** and Supplementary Figure S2). By contrast, members from *Clostridia* and *Bacteroidia* classes contributed to 23–52% of the annotated CAZymes across the metagenomes, where representation by these classes was between two and five times higher in poplar hydrolysate-fed cultures than in cellulose-fed ones. Moreover, CAZyme families that comprise plant polysaccharide-active enzymes (i.e., families GH2, GH3, GH5, GH9, GH43, GH51, and CE1) were most frequently assigned to either *Clostridia* or *Bacteroidia* (Supplementary Figure S2). In particular, GH5 sequences were most frequently assigned to *Clostridia* in poplar hydrolysate-fed cultures, whereas GH2, GH3, and GH43 were most frequently assigned to class *Bacteroidia* in poplar hydrolysate-fed cultures.

**FIGURE 2:**
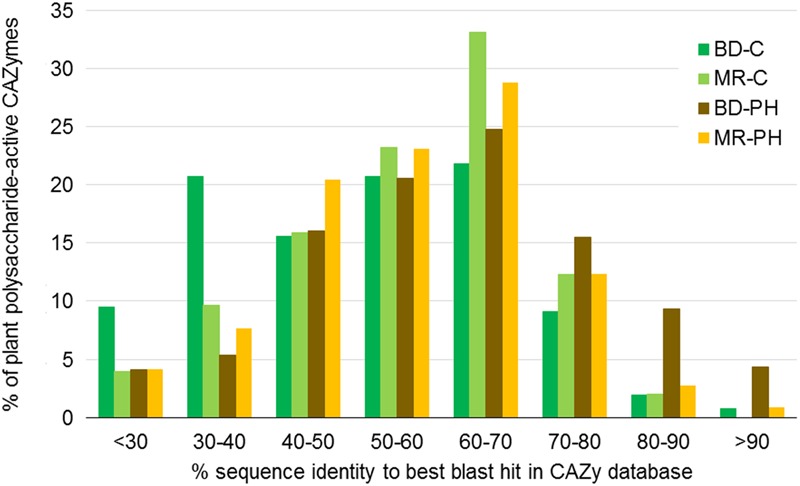
Percent identity between amino acid sequences in the CAZy database and CAZyme sequences predicted in beaver dropping (BD) and moose rumen (MR) microcosms enriched with cellulose (C) and poplar hydrolysate (PH). Percent identities correspond to best blast hits in the CAZy database, and were obtained for CAZyme sequences belonging to CAZyme families known to contain enzymes that act on plant cell wall carbohydrates (**Figure 4A**).

Considering all plant polysaccharides-degrading CAZymes predicted in each metagenome, more than half were less than 60% identical to the CAZyme amino acid sequences reported in the CAZy database (**Figure 2**). While percent identities varied depending on CAZyme family (Supplementary Figure S3), the most divergent sequences belong to families GH113 (on average 34% identical to their closest blast hits), PL22 (35%), GH5 (40%), GH74 (40%), and PL9 (43%).

**FIGURE 3:**
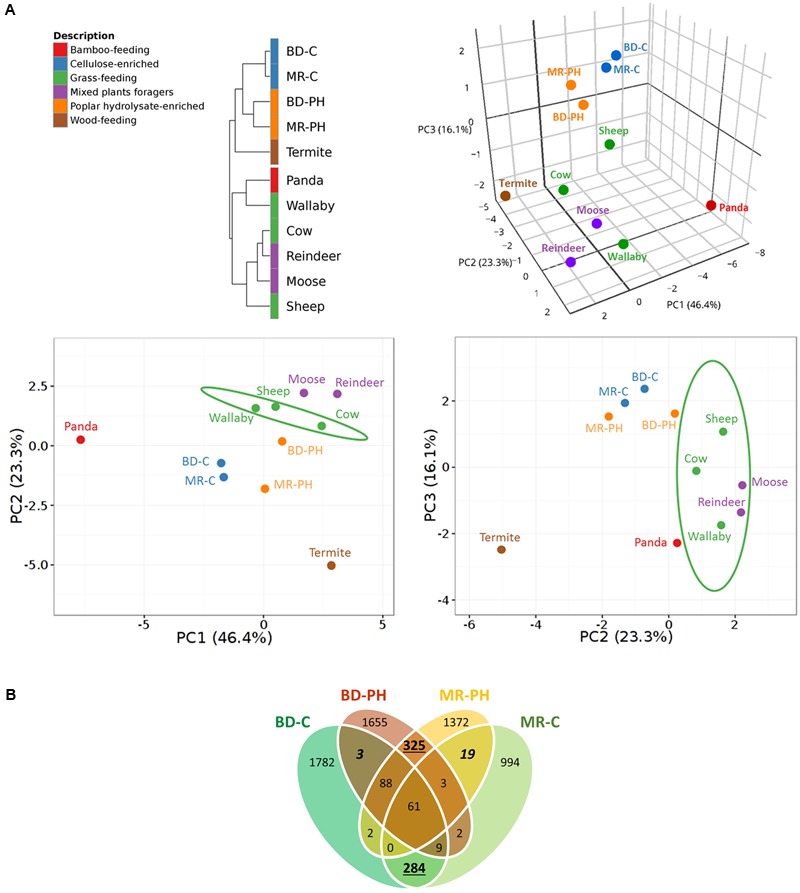
**(A)** Correlation clustering and PCA plots of CAZyme profiles encoded by metagenomes from lignocellulose degrading microbial communities. CAZyme families known to contain enzymes that act on plant cell wall carbohydrates were considered in the analysis. Public datasets included cow (Hess et al., 2011), moose (Svartström et al., 2017), panda (Zhu et al., 2011), reindeer (Pope et al., 2012), Saudi sheep (Al-Masaudi et al., 2017), termite (Warnecke et al., 2007), and wallaby (Pope et al., 2010). A 3D PCA plot is shown on the top right corner with the corresponding 2D PCA plots shown at the bottom; confidence intervals (95%) are indicated by the ellipses. **(B)** Venn diagram showing a greater overlap of unique CAZyme sequences in cultures fed with the same substrates (numbers underlined) than those that originate from the same inocula (numbers in italics).

Hierarchical clustering analysis of plant polysaccharide-active CAZyme families distinguished those reported in this study from those previously predicted from grass-feeding mammals or mixed plants foragers (**Figure 3A**). In particular, the distribution of the CAZyme families predicted in moose rumen enrichments differed from that recently reported for the moose rumen metagenome (Svartström et al., 2017), where highest contributing factors were attributed to relatively high abundance of CE4, GH94, and GH78 families, and low abundance of GH2, GH43 in the moose rumen enrichments (Supplementary Table S3). Consistent with substrate-induced convergence (Wong et al., 2016), long-term *ex situ* enrichment prior to metagenome sequencing also led to higher similarity of CAZyme distributions for cultures fed with the same carbon source (i.e., poplar hydrolysate or cellulose) as opposed to originating from the same environmental source (i.e., moose rumen or beaver droppings) (**Figure 3A**). The observed substrate-driven convergence of metagenomes was mostly attributed to higher relative abundances of GH2, GH3, GH43, CE1, CE4 in cultures enriched on poplar hydrolysate (Supplementary Table S3). At the same time, a greater overlap of unique CAZyme sequences was observed between cultures fed with the same substrate than those with the same inoculum (**Figure 3B**). It is also worthwhile to note that the plant polysaccharide-active CAZyme families from termite gut, albeit wood-feeding, do not cluster closely with those from the poplar hydrolysate enrichments due to the latter’s lower relative abundances of GH5, GH10, and GH94 (Supplementary Table S3). This likely reflects differences in the wood substrates consumed, as well as intrinsic differences in the gut microbiome of mammals and insects. Nonetheless, along PC2 where these metagenomes diverge the most, the PCA plot depicted a closer resemblance of CAZyme profiles microbiomes from termite gut and moose rumen samples enriched on poplar hydrolysate, than that between the former and the non-enriched moose rumen.

### Impact of Enrichment Substrate on Profiles of Predicted Plant–Polysaccharide Degrading CAZyme Sequences

**FIGURE 4:**
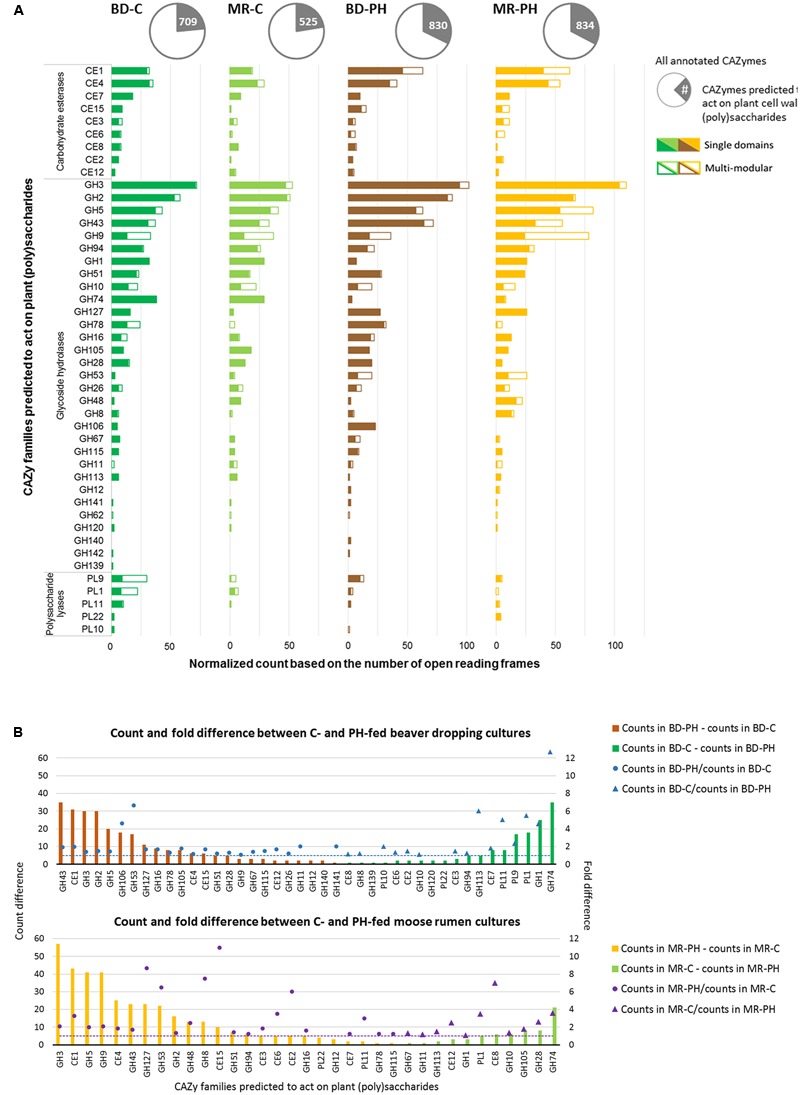
**(A)** Distribution of plant (poly)saccharide degrading-CAZyme families as single and multi-modular domains. **(B)** Normalized count and fold difference of CAZyme families predicted to act on plant polysaccharides between poplar hydrolysate (PH)- and cellulose (C)-fed beaver dropping (BD) and moose rumen (MR) cultures. Fold difference was only calculated for non-zero counts.

Metagenomes of cultures enriched on poplar hydrolysate yielded a higher proportion of predicted plant polysaccharides-active CAZymes (∼33%) compared to metagenomes of cultures enriched on cellulose (∼23%) (**Table 1** and **Figure 4A**). In particular, poplar hydrolysate-degrading communities were enriched in sequence counts from families GH3, GH5, GH43, CE1, and GH53 (**Figure 4A**). Additional substrate-induced differences were noted when considering moose rumen and beaver dropping enrichments separately. Beaver dropping samples enriched on poplar hydrolysate encoded higher counts of GH2 (1.5 times higher) and GH106 (4.6 times) than corresponding samples enriched on cellulose (**Figure 4B**). Meanwhile, moose rumen samples enriched on poplar hydrolysate encoded higher counts of GH9 (2 times higher), CE4 (1.8 times), GH127 (9 times), and CE15 (11 times) compared to corresponding samples enriched on cellulose (**Figure 4B**).

Carbohydrate-active enzymes families that were enriched through growth on poplar hydrolysate included those that comprise enzymes involved in plant polysaccharide deconstruction. For example, family GH43 includes enzymes that target arabinoxylan (Borsenberger et al., 2014; Mewis et al., 2016), family CE1 members were shown to deacetylate polymeric xylans (Neumuller et al., 2015; Mai-Gisondi et al., 2017), and family GH5 members include endoxylanases that targets xylans with or without methyl-glucuronic acid side chain (Gallardo et al., 2010), as well as enzymes that target cellulose and mannans (Aspeborg et al., 2012). Notably, enzymes belonging to families GH2 and GH3 were also abundant in the moose rumen microbiome, and predicted to participate in plant cell wall deconstruction (Svartström et al., 2017). Characterized CE15 members display 4-*O*-methyl-glucuronoyl methylesterase activity, which are thought to hydrolyze ester linkages that may form between hydroxyl groups in lignin and 4-*O*-methyl-D-glucuronic acid residues in glucuronoxylans that dominate in hardwood fiber (Biely et al., 2015; Biely, 2016). Recently, a marine bacterial CE15 enzyme predicted to act on alginates was also reported, suggesting a broader substrate range for this CE family (De Santi et al., 2016; Agger et al., 2017). On the other hand, GH127 enzymes typically contain β-L-arabinofuranosidases that have been shown to target plant cell wall glycoproteins, such as extensin (Fujita et al., 2011). By contrast, between 3 and 13 times more GH74 sequences were identified in cellulose-fed enrichments compared to corresponding cultures enriched on poplar hydrolysate, fitting with the endoglucanase activity reported for this CAZyme family (Song et al., 2017). Similarly, 4.6 times more GH1 sequences were identified in the beaver dropping culture enriched on cellulose than that on poplar hydrolysate; characterized bacteria members from this family large act as β-glucosidases that hydrolyze cellobiose and soluble cellodextrins to glucose (Singhania et al., 2013). Interestingly, families capable of pectin degradation (PL1, PL9) were also found at higher abundances in the cellulose-fed beaver dropping culture than that fed with poplar hydrolysate.

Carbohydrate-binding modules can impact enzyme performance through targeting catalytic modules to polysaccharide substrates, and in some cases promote non-hydrolytic fiber disruption (Boraston et al., 2004; Gourlay et al., 2012); accordingly, CAZymes with cognate CBMs were also predicted from each metagenome sequence. About 20% of the sequences predicted to encode plant polysaccharides degrading enzymes (i.e., 669 sequences) were predicted to form multi-domain proteins (Supplementary Table S4). Most frequent domain organizations included CBMs, such as CBM48-GH13_9 (7–8% in cellulose enrichments), GH9-CBM3-CBM3 (∼6% in moose rumen samples enriched on poplar hydrolysate), and CBM50-CBM50-GH18 (∼6% in beaver dropping and moose rumen samples enriched on poplar hydrolysate and cellulose, respectively). While CBM48-GH13 is a documented architecture for starch-degrading enzymes (Machovic and Janecek, 2008), the modular architecture GH9-CBM3-CBM3 was previously only reported as a non-cellulosomal enzyme encoded by *Clostridium thermocellum* (Anitori, 2012). CBM50-CBM50-GH18, like other GH18 chitinases with multiple CBM50 domains, was predicted to bind peptidoglycan-like and chitin-derived oligosaccharides (Bateman and Bycroft, 2000). Contrary to findings recently reported for the moose rumen microbiome (Svartström et al., 2017), the multi-modular enzymes comprising CBM50 and GH23 or GH73 were identified at low abundance (<3.5%) in the enriched moose rumen metagenomes reported herein.

In addition to multi-modular CAZymes comprising CBMs, those comprising potential cellulosomal subunits were also predicted. Cellulosomes are cell-associated multi-enzyme complexes that are produced by certain anaerobic bacteria to promote polysaccharide degradation (White et al., 2014; Artzi et al., 2017; Smith et al., 2017). When expressed as cell-bound cellulosomes (opposed to cell-free cellulosomes), the primary scaffoldins are connected through type II dockerin-cohesin interactions to specialized anchoring scaffoldins, which contain peptidoglycan-binding S-layer homology modules that anchor to the cell surface. Type I interactions, on the other hand, occur between the dockerin-containing enzymatic subunits and the cohesins on the primary scaffoldins.

**FIGURE 5:**
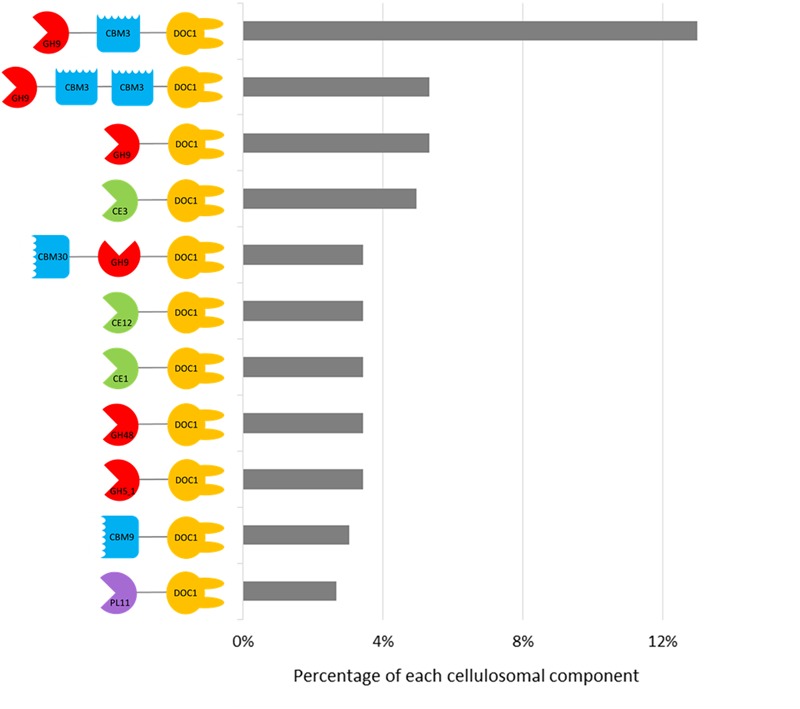
Catalog of domain architectures of top 11 abundant CAZy-dockerins in the cellulose- and poplar hydrolysate-fed microbial enrichments from beaver droppings and moose rumen. CBM, carbohydrate-binding module; CE, carbohydrate esterase; DOC1, type 1 dockerin; GH, glycoside hydrolases; PL, polysaccharide lyase.

For all enrichments, approximately twice the number of dockerins compared to cohesins were predicted (**Table 1**), and 56% of dockerins were appended to CAZyme sequences (Supplementary Table S5). The most frequently occurring CAZyme module was family GH9 (∼29%), followed by GH5 (∼11%), CE3 (∼8%), GH43 (∼7%), and GH3 (∼6%) (**Figure 5** and Supplementary Table S4). And unlike the recent study of the moose rumen metagenome, the recurrent GH13 appended-dockerins (Svartström et al., 2017) were not identified in the current moose rumen metagenomes, likely due to their long enrichment on cellulosic carbon sources. Other common components of cellulosome systems (Artzi et al., 2017), such as GH10 (∼4%), GH11 (∼4%), and GH48 (∼4%) were also identified in the metagenomes of both moose rumen and beaver dropping enrichments, albeit at a lower abundance. Notably, the identification of few sequences containing a GH48 module and the approximately nine-fold higher number of those containing a GH9 module is consistent with the earlier analyses of cellulose-degrading anaerobic bacteria that generate high levels of a single GH48 and diverse GH9 enzymes with potential synergistic action (Morag et al., 1991; Ravachol et al., 2014; Artzi et al., 2015).

### Predicted Polysaccharide Utilization Loci (PULs)

**FIGURE 6:**
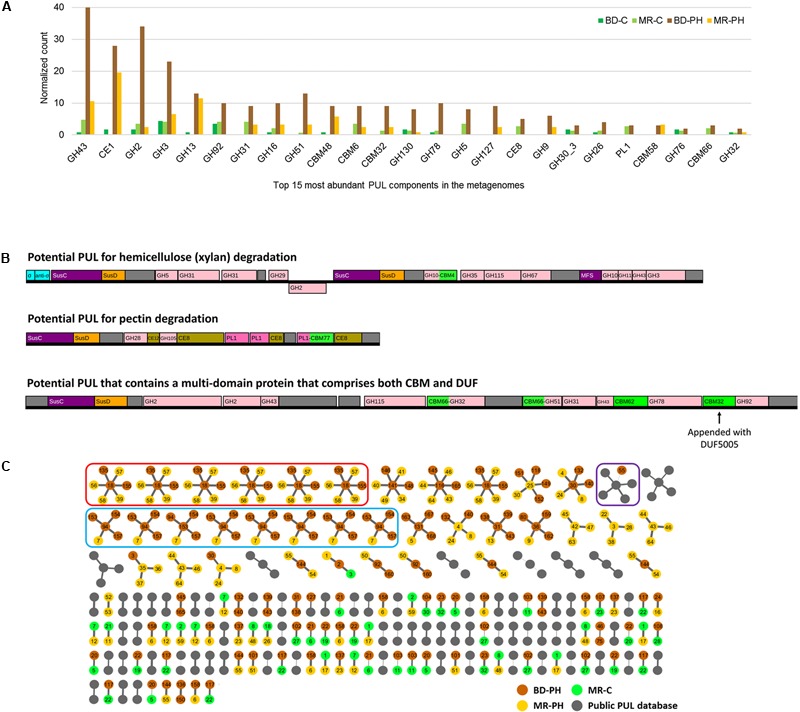
**(A)** Top 15 most abundant CAZyme families identified in predicted PULs from cellulose (C)- and poplar hydrolysate (PH)-fed microbial enrichments of beaver droppings and moose rumen. **(B)** Examples of predicted PULs from beaver droppings enriched on poplar hydrolysate (BD-PH). **(C)** Similarity-based clustering (≥70%) of proteins with unknown function positioned in PULs identified herein and listed in the public PUL database (http://www.cazy.org/PULDB/). Each cluster contains a central node that denotes the representative protein with unknown function (defined by the longest length) and connected nodes that represent a protein with unknown function that is ≥70% identical to the representative sequence (see the section “Materials and Methods”). PUL identifiers are shown on each node; the thickness of the edges correlates to percent identity between sequences. Circled in red and blue are proteins with unknown functions that are ≥95% identical to one another; the architecture of PULs circled in red is identical, whereas those circled in blue share a common central architecture but differ at flanking regions. Circled in purple is the only cluster that comprises proteins with unknown function from both PULs predicted herein and those from the public PUL database.

As summarized above, PULs have emerged as especially fruitful regions within genomic sequences for enzyme discovery (Larsbrink et al., 2016). Herein, 416 PULs were predicted (Supplementary Figure S4), where the normalized number predicted from poplar hydrolysate-fed microcosms of beaver droppings was 4.5 times that predicted from cellulose-fed microcosms. Consistent with the overall distribution of predicted CAZyme sequences, those belonging to families CE1, GH3, and GH43, were most frequently identified in the predicted PULs (**Figure 6A** and Supplementary Table S6). Moreover, PULs comprising members of families GH127 and GH9 were exclusively identified in metagenomes from cultures enriched on poplar hydrolysate. Based on the family composition of a given PUL, substrate category of the PUL-encoded enzymes can be inferred. For example, **Figure 6B** illustrates PULs that potentially target xylan and pectin based on the established activities of the CAZyme families. Sequences annotated as unknown may include novel enzyme functions, for instance as shown recently in the case of the type II rhamnogalacturonan PUL of *B. thetaiotaomicron* (Ndeh et al., 2017).

Of note, 620 sequences annotated as proteins with unknown function (with lengths ranging from 32 to 1,320 amino acids) were identified in all candidate PULs (Supplementary Figure S5A). Among these, eight sequences annotated as proteins with unknown function were found to comprise a CBM from family CBM32, CBM35, CBM51, or CBM66. In an effort to prioritize additional sequences for future characterization, a clustering network diagram was generated to uncover protein sequences with unknown function that reoccurred in the predicted PULs. However, little similarity was revealed between such sequences from PULs predicted herein and those reported in the public PULDB (Supplementary Figure S5B). In fact, only one such sequence from beaver droppings enriched on poplar hydrolysate was ≥70% identical to those annotated in the PULDB, and the few sequences with unknown function that did cluster typically originated from PULs with similar architecture (**Figure 6C** and Supplementary Figure S4).

### Predicted Multi-Modular Proteins – An Additional Source of Yet Unknown Carbohydrate-Active Proteins

A second approach to assist the discovery of potentially new CAZyme families considered multi-modular proteins predicted to comprise a DUF, or a sequence of unknown function, appended to a CBM or dockerins (cellulosomal subunit).

**FIGURE 7:**
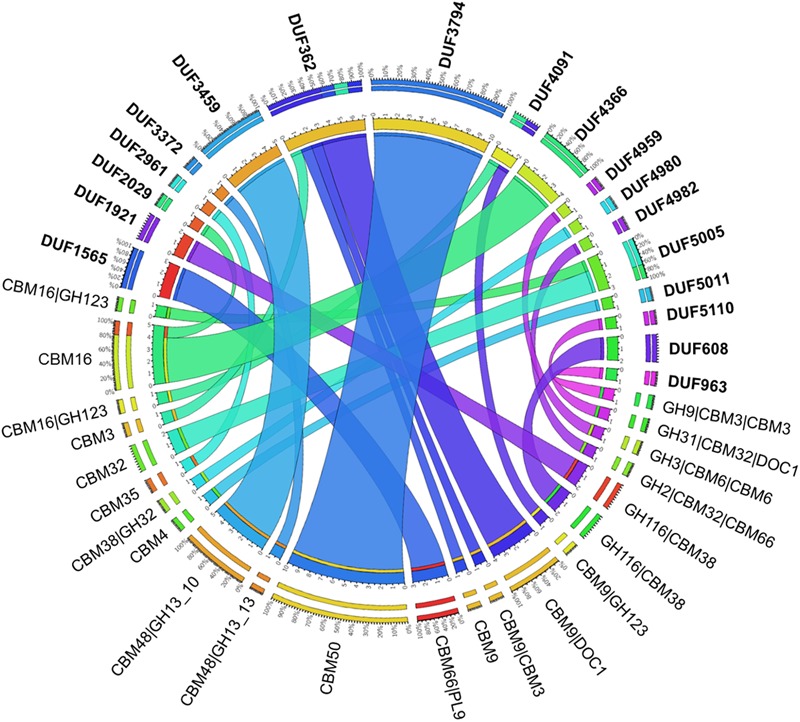
Carbohydrate-active proteins with domains of unknown functions (DUFs) identified in the metagenomes. In bold are the DUFs that are identified with various CBMs and CAZymes as shown via the connections color-coded in accordance to the DUFs. The number and corresponding percentage of each combination of modules are shown in the outer scale.

Considering all four metagenome sequences reported herein, 62 DUFs were identified that co-occurred with a CBM (**Figure 7** and Supplementary Figure S6). The most frequent organizations were: DUF3794-CBM50 (10 identified), DUF362-CBM9-DOC1 (4 identified), DUF4366-CBM16 (4 identified), which were identified in all four metagenomes; five DUF3459-CBM48-GH13_10 sequences were also identified in the metagenome of beaver droppings enriched on poplar hydrolysate (Supplementary Figure S6). As shown herein and also described in the Pfam database (Finn et al., 2016), DUF3794 was often found in association with CBM50. On the other hand, DUF362 is often present in proteins with domains that bind to iron–sulfur clusters, and its coexistence with CBM9_1 in an uncharacterized protein from soil bacteria *Sorangium cellulosum* was previously observed (UniProt entry S4XJL8). The structure of DUF3459 has been determined (UniProt entries B2IUW9, Q9RX51, Q8ZPF0, Q8P5I2, Q2PS28, M1E1F6, M1E1F3, H3K096), and as observed here, was previously shown to be part of multi-modular proteins comprising GH13 and CBM48 domains (UniProt entries W6LS46, R4KHQ4, C7RTS8). Although not frequently observed, one PUL identified in the poplar hydrolysate-fed beaver droppings culture contained DUF5005 appended to a predicted CBM32 (**Figure 6B**).

Of the predicted dockerin sequences, 44% lacked known appended CAZyme modules. Similar to previous reports (Finn et al., 2016), a few dockerin sequences were predicted to have appended DUF (i.e., DUF362, DUF1533, and DUF3237); however, the majority were not annotated as containing modules or domains functionally attributed to cellulosomes. In many cases, this could reflect sequence gaps due to incomplete metagenome assembly; however, it is also conceivable that dockerin–cohesin proteins might in fact participate in other biological functions, as suggested by a phylogenetically distinct group of cohesins discovered in the cow rumen metagenome (Bensoussan et al., 2017).

## Conclusion

Given their natural dietary habits, the Canadian beaver and North American moose have likely evolved digestive microbiomes with the ability to degrade diverse wood polysaccharides. To identify enzymes that may be most relevant to wood fiber bioprocessing, corresponding microbial communities were enriched for approximately 3 years on comparatively complex (poplar hydrolysate) and defined (microcrystalline cellulose) carbon sources. Enrichment led to substrate-induced convergence of CAZyme profiles, which significantly narrowed the number of CAZyme families and corresponding members that could be targeted and tested for improved enzymatic conversion of wood fiber. For example, in addition to families GH2, GH3, GH5, and GH43 which previous reports have also identified, GH127 and CE15 may be especially relevant for the anaerobic conversion of pretreated wood fiber. Protein sequences containing both a CBM and a DUF, as well as proteins with unknown function yet having signals for secretion and position within PULs, were also identified and may facilitate the discovery of new CAZyme activities. Proteomic analysis of secretomes from the enrichment cultures prepared herein will provide an additional filter for protein selection and characterization. In the interim, however, enrichment followed by comparative metagenomics sufficiently narrowed protein lists of primary interest, enabling direct recombinant production and characterization.

## Author Contributions

MW performed the sequence analyses, data interpretation, and compiled the manuscript. WW maintained the enrichment cultures, prepared DNA samples for sequencing, and contributed to data interpretation. MC and FR contributed to data interpretation. BH, NT, VL, and PL contributed to the search and annotation of CAZyme modules and PULs, as well as data interpretation. EM and EE conceived and coordinated the study. All authors contributed to the revision of manuscript and approved the final version.

## Conflict of Interest Statement

The authors declare that the research was conducted in the absence of any commercial or financial relationships that could be construed as a potential conflict of interest.

## Footnotes

1 www.cazy.org

2 http://www.cazy.org/PULDB/

## Abbreviations

AA: auxiliary activity
ABySS: Assembly By Short Sequences
CAZyme: carbohydrate-active enzyme
CBM: carbohydrate-binding module
CE: carbohydrate esterase
COD: chemical oxygen demand
DUF: domain of unknown function
ECF: extracytoplasmic function
GH: glycoside hydrolase
GT: glycosyltransferase
NCBI-NR: National Center for Biotechnology Information Non-redundant
NKD: normalized kmer depths
ORF: open-reading frame
PCA: principal component analysis
PUL: polysaccharide utilization locus
RPKM: reads per kilobase per million mapped reads
