# Supplementary material for: Comparative Metagenomics of Cellulose- and Poplar Hydrolysate-Degrading Microcosms from Gut Microflora of the Canadian Beaver (Castor canadensis) and North American Moose (Alces americanus) after Long-Term Enrichment
Source: Front Microbiol. 2017 Dec 20;8:2504. doi: 10.3389/fmicb.2017.02504 (PMC5742341; doi:10.3389/fmicb.2017.02504)
Supplement: Supplementary file 1 [file Presentation_1.pdf]

Beaver dropping microbial cultures enriched with cellulose (BD-C)

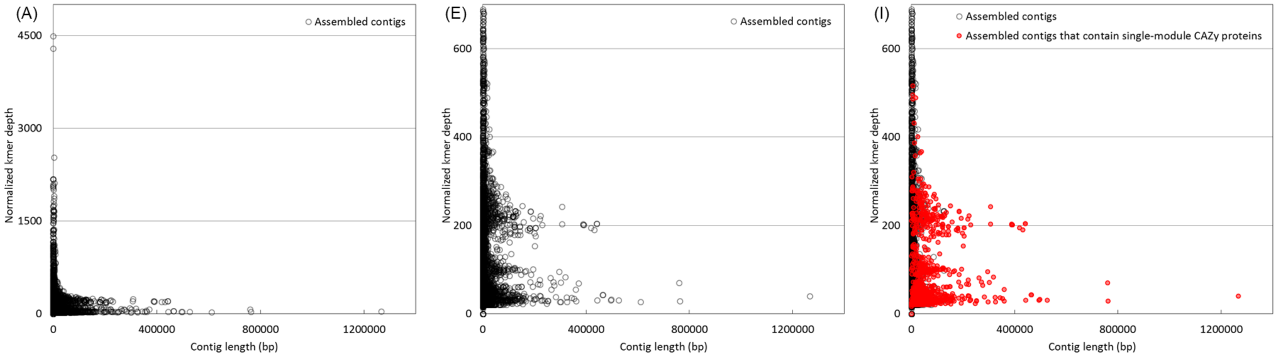

Beaver dropping microbial cultures enriched with poplar hydrolysate (BD-PH)

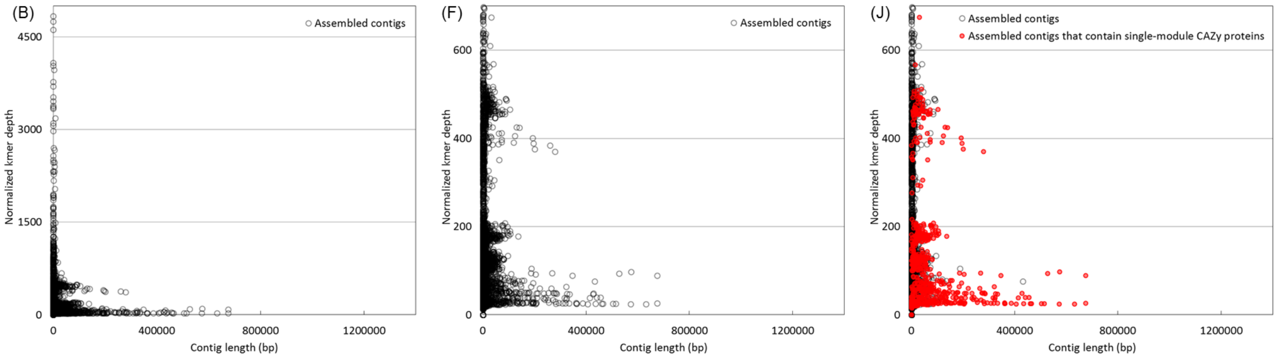

Moose rumen microbial cultures enriched with cellulose (MR-C)

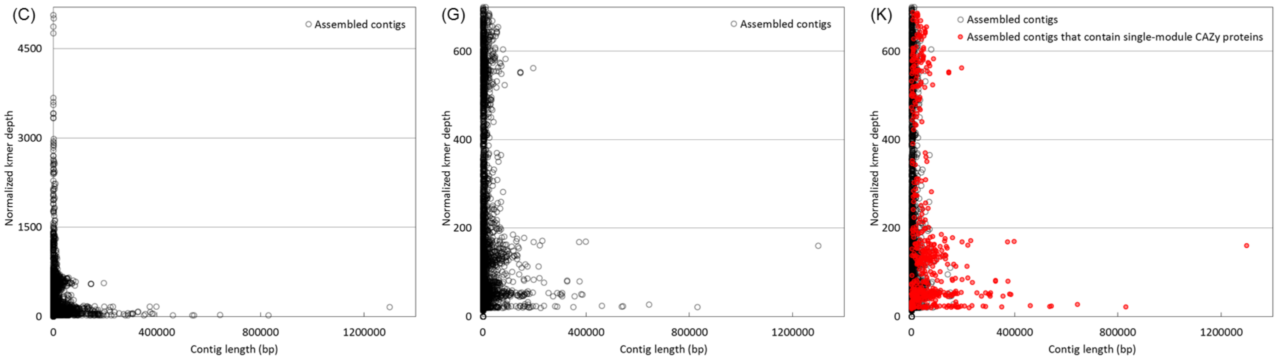

Moose rumen microbial cultures enriched with poplar hydrolysate (MR-PH)

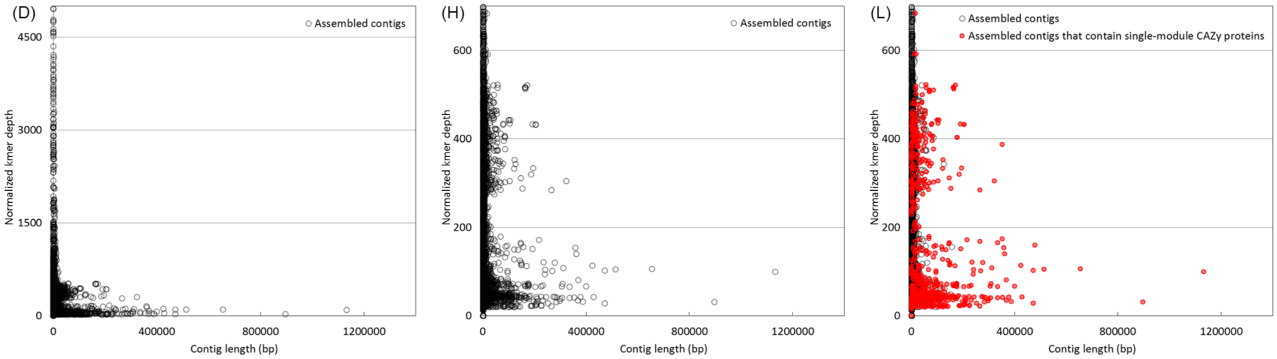

BD-C

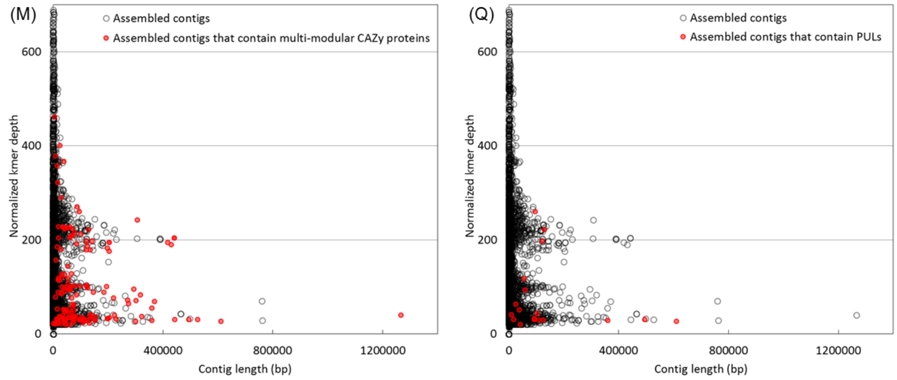

BD-PH

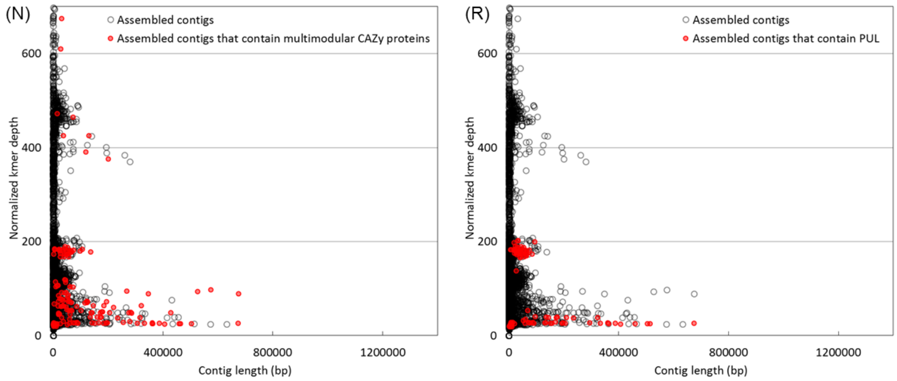

MR-C

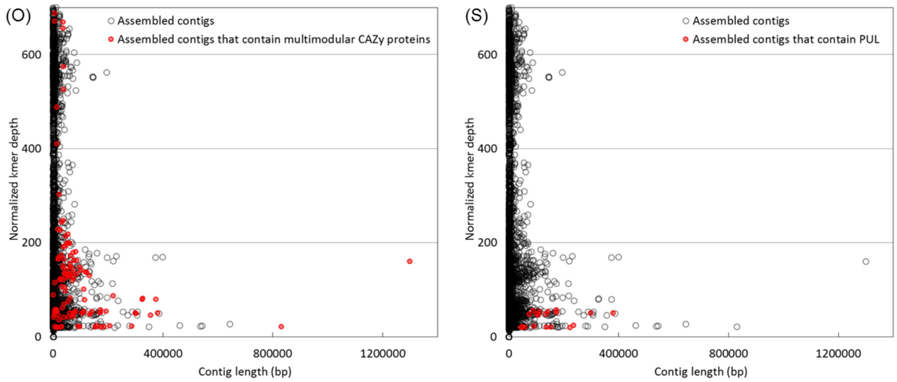

MR-PH

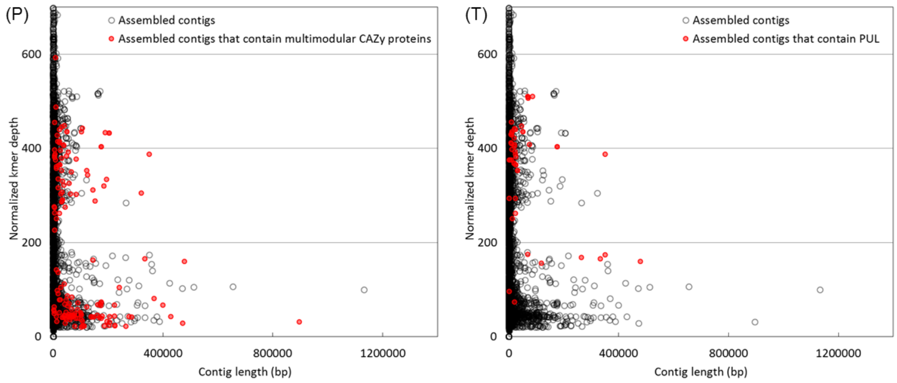

Figure S1 Contig profiles depicting the distribution of contig lengths at different normalized kmer depths for (A-D) all assembled contigs and (E-H) those within kmer depths where longer contigs locate, and with contigs that contain (I-L) single module (M-P) and multi-modular CAZymes, as well as (Q-T) PULs highlighted in red.

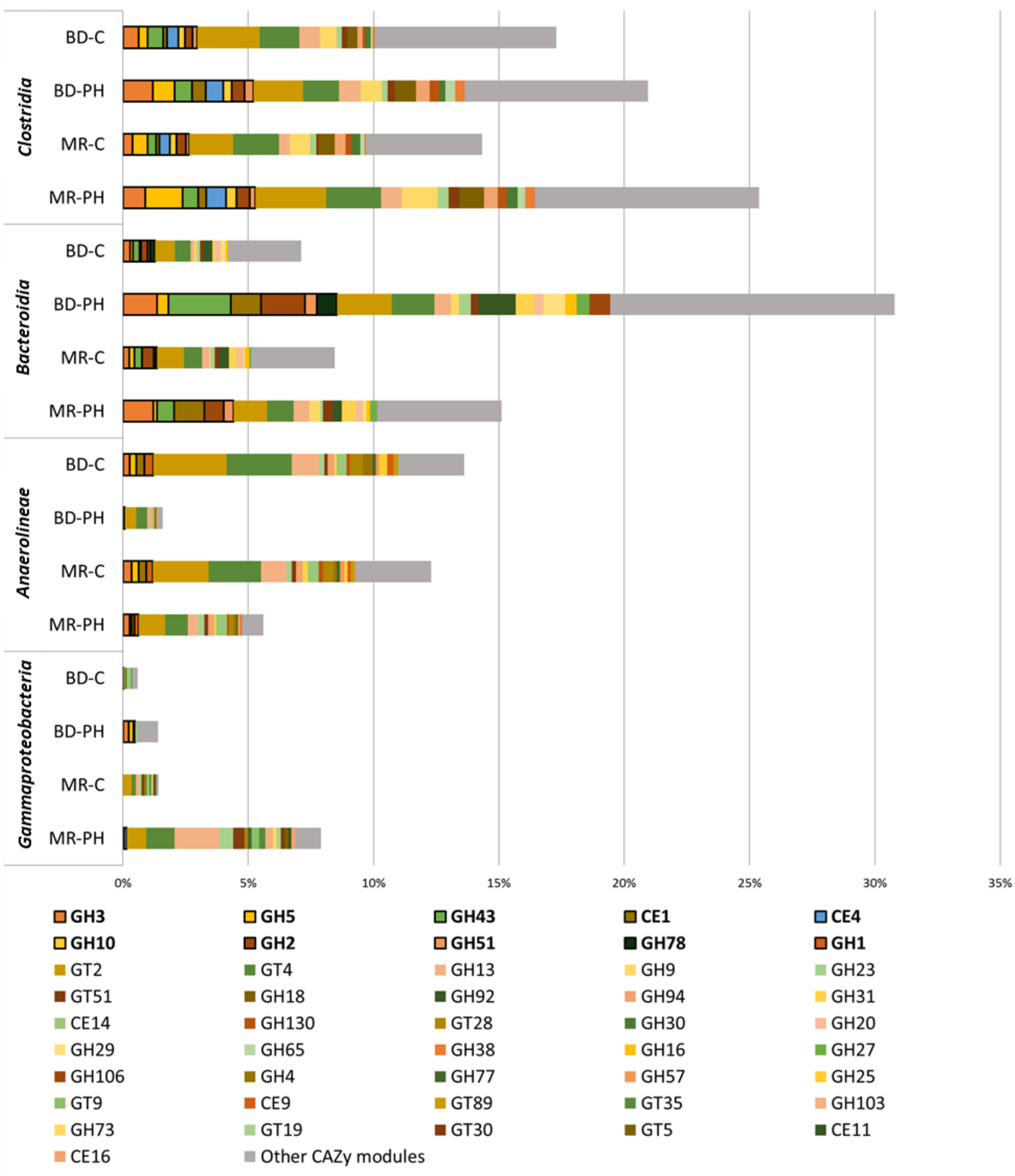

Figure S2 Top 20 CAZyme families assigned to most abundant identified classes: *Clostridia*, *Bacteroidia*, *Anaerolinease*, and *Gammaproteobacteria*. CAZyme families predicted to act on plant polysaccharides are outlined in black.

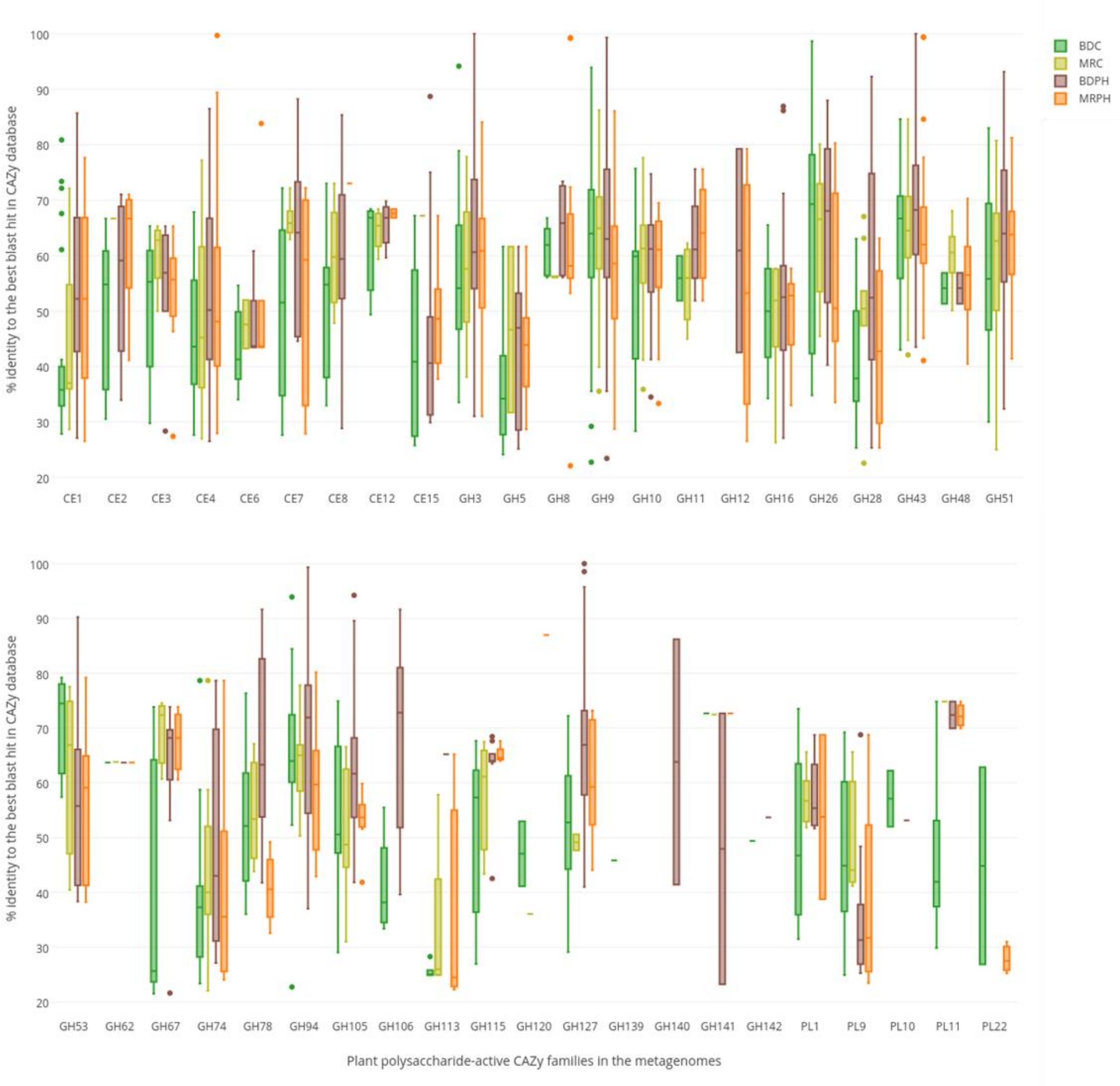

Figure S3 Distribution of percentage identities of the plant polysaccharide-active CAZyme families in beaver dropping (BD) and moose rumen (MR) microcosms enriched with cellulose (C) and poplar hydrolysate (PH) with respective best blast hits in the CAZy database.

Sample	PUL Nº	Modularity
BD-C	Predicted 1	ECF-σ ▶ SusC ▶ SusD ▶
BD-C	Predicted 2	HTCS ▶ SusC ▶ SusD ▶ GH32 ▶ unk ▶ MFS ▶ unk ▶ CE7 ▶
BD-C	Predicted 3	GH2 ▶ unk ▶ Anti-σ ▶ SusC ▶ SusD ▶ unk ▶ ECF-σ ▶
BD-C	Predicted 4	unk ▶ GH130 ▶ SusC ▶ SusD ▶ unk ▶ unk ▶ GH26 ▶
BD-C	Predicted 5	ECF-σ ▶ Anti-σ ▶ SusC ▶ SusD ▶ unk ▶ GH76 ▶ GH76 ▶ GH125 ▶
BD-C	Predicted 6	ECF-σ ▶ Anti-σ ▶ SusC ▶ SusD ▶ unk ▶ unk ▶
BD-C	Predicted 7	SusC ▶ SusD ▶
BD-C	Predicted 8	ECF-σ ▶ Anti-σ ▶ SusC ▶ SusD ▶
BD-C	Predicted 9	SusC ▶ SusD ▶
BD-C	Predicted 10	SusC ▶ SusD ▶
BD-C	Predicted 11	SusC ▶ SusD ▶ SusC ▶ SusD ▶ SusC ▶ SusD ▶
BD-C	Predicted 12	ECF-σ ▶ Anti-σ ▶ SusC ▶ SusD ▶ unk ▶ GH63 ▶ unk ▶
BD-C	Predicted 13	SusC ▶ SusD ▶
BD-C	Predicted 14	SusC ▶ SusD ▶ Pept_SC ▶
BD-C	Predicted 15	ECF-σ ▶ Anti-σ ▶ SusC ▶ SusD ▶
BD-C	Predicted 16	ECF-σ ▶ Anti-σ ▶ SusC ▶ SusD ▶ unk ▶ GH2|GH130 ▶
BD-C	Predicted 17	ECF-σ ▶ Anti-σ ▶ SusC ▶ SusD ▶ unk ▶
BD-C	Predicted 18	◀ ECF-σ Anti-σ ▶ SusC ▶ SusD ▶
BD-C	Predicted 19	◀ unk ◀ GH92 SusC ▶ SusD ▶
BD-C	Predicted 20	SusC ▶ SusD ▶
BD-C	Predicted 21	◀ GH78 ◀ HTCS unk ▶ SusC ▶ SusD ▶ GH3 ▶ CE1 ▶ CE1 ▶
BD-C	Predicted 22	SusC ▶ SusD ▶ unk ▶ unk ▶ unk ▶ unk ▶ unk ▶ unk ▶
BD-C	Predicted 23	ECF-σ ▶ Anti-σ ▶ SusC ▶ SusD ▶
BD-C	Predicted 24	SusC ▶ SusD ▶ GH30_3 ▶ unk ▶ GH30_3 ▶ GH3 ▶
BD-C	Predicted 25	ECF-σ ▶ Anti-σ ▶ SusC ▶ SusD ▶ GH92 ▶ GH92 ▶ GH92 ▶
BD-C	Predicted 26	ECF-σ ▶ Anti-σ ▶ SusC ▶ SusD ▶ GH109 ▶
BD-C	Predicted 27	SusC ▶ SusD ▶ unk ▶ CBM48|GH13 ▶

BD-C	Predicted 28	SusC ▶ SusD ▶
BD-C	Predicted 29	SusC ▶ SusD ▶ unk ▶ GH3 ▶ unk ▶ GH43_28 ▶ unk ▶
BD-C	Predicted 30	SusC ▶ SusD ▶
BD-C	Predicted 31	◀ ECF-σ Anti-σ ▶ SusC ▶ SusD ▶ unk ▶ unk ▶ unk ▶ GT2 ▶ unk ▶ MFS ▶ unk ▶ unk ▶ unk ▶ unk ▶
BD-C	Predicted 32	GH3 ▶ unk ▶ SusC ▶ SusD ▶ unk ▶ GH16 ▶ GH3 ▶
BD-C	Predicted 33	SusC ▶ SusD ▶
BD-PH	Predicted 1	SusC ▶ SusD ▶
BD-PH	Predicted 2	SusC ▶ SusD ▶ unk ▶
BD-PH	Predicted 3	SusC ▶ SusD ▶ unk ▶
BD-PH	Predicted 4	SusC ▶ SusD ▶ unk ▶
BD-PH	Predicted 5	◀ HTCS SusC ▶ SusD ▶ unk ▶ unk ▶ GH43 ▶ GH127 ▶ unk ▶ unk ▶
BD-PH	Predicted 6	◀ GH93 ◀ GH43 SusC ▶ SusD ▶ GH43 ▶ HTCS ▶ GH2 ▶ GH93 ▶ GH43 ▶
BD-PH	Predicted 7	ECF-σ ▶ CE1 ▶ Anti-σ ▶ SusC ▶ SusD ▶ unk ▶ unk ▶ GH5_36 ▶ GH28 ▶ unk ▶ unk ▶ unk ▶
BD-PH	Predicted 8	GH78 ▶ SusC ▶ SusD ▶ unk ▶ GH2 ▶ unk ▶ unk ▶ unk ▶ MFS ▶ GH36 ▶ GH36 ▶ unk ▶
BD-PH	Predicted 9	ECF-σ ▶ Anti-σ ▶ SusC ▶ SusD ▶ GH2 ▶ GH105 ▶
BD-PH	Predicted 10	ECF-σ ▶ Anti-σ ▶ SusC ▶ SusD ▶
BD-PH	Predicted 11	Anti-σ ▶ SusC ▶ SusD ▶ unk ▶ GH29 ▶ unk ▶ unk ▶ unk ▶ GH3 ▶ unk ▶ unk ▶ unk ▶ unk ▶ GH95 ▶ unk ▶ unk ▶
BD-PH	Predicted 12	ECF-σ ▶ Anti-σ ▶ SusC ▶ SusD ▶ unk ▶ unk ▶ unk ▶
BD-PH	Predicted 13	SusC ▶ SusD ▶ SusC ▶ SusD ▶ Pept_SC ▶ Pept_CA ▶ Pept_MH ▶ Pept_MH ▶ unk ▶ CE1 ▶ unk ▶
BD-PH	Predicted 14	SusC ▶ SusD ▶ unk ▶
BD-PH	Predicted 15	SusC ▶ SusD ▶ unk ▶ unk ▶
BD-PH	Predicted 16	unk ▶ unk ▶ SusC ▶ SusD ▶
BD-PH	Predicted 17	unk ▶ SusC ▶ SusD ▶ unk ▶

BD-PH	Predicted 18	MFS ▶ SusC ▶ SusD ▶ unk ▶ unk ▶ CBM58|GH13 ▶ unk ▶ GH13 ▶ GH13 ▶ unk ▶ unk ▶ unk ▶ unk ▶
BD-PH	Predicted 19	SusC ▶ SusD ▶ unk ▶ unk ▶
BD-PH	Predicted 20	SusC ▶ SusD ▶ unk ▶ unk ▶ unk ▶ unk ▶
BD-PH	Predicted 21	SusC ▶ SusD ▶ unk ▶ GH28 ▶ CE12 ▶ GH105 ▶ CE8 ▶ PL1 ▶ PL1 ▶ CE8 ▶ unk ▶ PL1|CBM77 ▶ CE8 ▶ unk ▶
BD-PH	Predicted 22	SusC ▶ SusD ▶ unk ▶ unk ▶ GH26 ▶ GH26 ▶ GH130 ▶ GH27 ▶ GH5_25 ▶ MFS ▶ unk ▶ GH5_2 ▶ unk ▶ GH3 ▶ ◀ unk GH20 ▶ unk ▶ GH20 ▶
BD-PH	Predicted 23	unk ▶ GH76 ▶ SusC ▶ SusD ▶ unk ▶
BD-PH	Predicted 24	unk ▶ SusC ▶ SusD ▶
BD-PH	Predicted 25	SusC ▶ SusD ▶
BD-PH	Predicted 26	◀ GH5_36 ◀ GH106 ◀ unk ◀ Pept_CA unk ▶ ◀ GH105 ◀ unk unk ▶ SusC ▶ SusD ▶ unk ▶
BD-PH	Predicted 27	SusC ▶ SusD ▶
BD-PH	Predicted 28	SusR ▶ unk ▶ unk ▶ SusC ▶ SusD ▶ GH99 ▶ GH97 ▶
BD-PH	Predicted 29	SusC ▶ SusD ▶
BD-PH	Predicted 30	SusC ▶ SusD ▶ GH43 ▶ CE1|CE6 ▶ GH9 ▶ CE1|CBM48|CBM32|GH43 ▶ CE1|CBM48 ▶ GH51 ▶ CE1 ▶ CE1 ▶ unk ▶ CBM6|GH43 ▶ unk ▶
BD-PH	Predicted 31	SusC ▶ SusD ▶ unk ▶ ◀ GH2 ◀ GH53|GH53 ◀ unk
BD-PH	Predicted 32	ECF-σ ▶ Anti-σ ▶ SusC ▶ SusD ▶ unk ▶ MFS ▶ unk ▶ GH9|CE4 ▶ unk ▶ GT2 ▶ unk ▶ unk ▶ MFS ▶
BD-PH	Predicted 33	SusC ▶ SusD ▶ SusD ▶ Sulf_1 ▶ Sulf_1 ▶ GH33 ▶
BD-PH	Predicted 34	SusC ▶ SusD ▶ CBM67 ▶
BD-PH	Predicted 35	◀ HTCS SusC ▶ SusD ▶
BD-PH	Predicted 36	SusC ▶ SusD ▶ GH2 ▶ GH43 ▶ unk ▶ GH35 ▶ HTCS ▶
BD-PH	Predicted 37	HTCS ▶ GH2 ▶ SusC ▶ SusD ▶ unk ▶ GH88 ▶ ◀ GH92
BD-PH	Predicted 38	SusC ▶ SusD ▶
BD-PH	Predicted 39	unk ▶ unk ▶ SusC ▶ SusD ▶ unk ▶ unk ▶ unk ▶ GH43 ▶ GH97 ▶ GH92 ▶ unk ▶ ◀ GH43 ◀ GH3
BD-PH	Predicted 40	SusC ▶ SusD ▶

BD-PH	Predicted 41	◀ HTCS GH130 ▶ MFS ▶ unk ▶ SusC ▶ SusD ▶ SusC ▶ SusD ▶
BD-PH	Predicted 42	unk ▶ SusC ▶ SusD ▶ unk ▶ GH53 ▶ GH2 ▶ unk ▶
BD-PH	Predicted 43	SusC ▶ SusD ▶ unk ▶ unk ▶ ◀ unk ◀ GH95 GH43 ▶ unk ▶ GH3 ▶ GH92 ▶ GH95|GH43 ▶ GH3 ▶ CBM32|GH43 ▶ GH92 ▶ GH2 ▶ GH3 ▶ ◀ GH2
BD-PH	Predicted 44	ECF-σ ▶ Anti-σ ▶ SusC ▶ SusD ▶ unk ▶ GH55 ▶ GH9 ▶ GH55 ▶ GH55 ▶ GH50 ▶ unk ▶ unk ▶ GH32 ▶
BD-PH	Predicted 45	SusC ▶ SusD ▶ unk ▶ GH16 ▶ GH3 ▶
BD-PH	Predicted 46	ECF-σ ▶ Anti-σ ▶ SusC ▶ SusD ▶ GH105 ▶ unk ▶
BD-PH	Predicted 47	ECF-σ ▶ Anti-σ ▶ SusC ▶ SusD ▶
BD-PH	Predicted 48	unk ▶ SusC ▶ SusD ▶ unk ▶
BD-PH	Predicted 49	SusC ▶ SusD ▶ unk ▶ unk ▶ unk ▶ GH130 ▶ Pept_SE ▶ unk ▶ unk ▶
BD-PH	Predicted 50	unk ▶ SusC ▶ SusD ▶
BD-PH	Predicted 51	ECF-σ ▶ Anti-σ ▶ SusC ▶ SusD ▶ unk ▶ unk ▶ GH88 ▶ unk ▶ PL17 ▶ PL8 ▶
BD-PH	Predicted 52	ECF-σ ▶ Anti-σ ▶ SusC ▶ SusD ▶ unk ▶
BD-PH	Predicted 53	unk ▶ unk ▶ unk ▶ unk ▶ GH36 ▶ SusC ▶ SusD ▶ unk ▶ unk ▶
BD-PH	Predicted 54	SusC ▶ SusD ▶ unk ▶
BD-PH	Predicted 55	unk ▶ SusC ▶ SusD ▶ unk ▶
BD-PH	Predicted 56	SusC ▶ SusD ▶ unk ▶ unk ▶ GH26|CBM35 ▶ GH26 ▶ GH130 ▶ MFS ▶ unk ▶ unk ▶
BD-PH	Predicted 57	unk ▶ SusC ▶ SusC ▶ SusD ▶ SusD ▶
BD-PH	Predicted 58	SusC ▶ SusD ▶ unk ▶
BD-PH	Predicted 59	SusC ▶ SusD ▶
BD-PH	Predicted 60	◀ ECF-σ Anti-σ ▶ SusC ▶ SusD ▶ unk ▶
BD-PH	Predicted 61	ECF-σ ▶ Anti-σ ▶ SusC ▶ SusD ▶ unk ▶ MFS ▶ GH51 ▶ GH51 ▶ Sulf_1 ▶ Pept_SC ▶
BD-PH	Predicted 62	SusC ▶ SusD ▶ unk ▶
BD-PH	Predicted 63	SusC ▶ SusD ▶ unk ▶ unk ▶ unk ▶
BD-PH	Predicted 64	SusC ▶ SusD ▶

BD-PH	Predicted 65	ECF-σ ▶ Anti-σ ▶ SusC ▶ SusD ▶ unk ▶ CBM32|GH2 ▶ ◀ unk ◀ GH3 |Pept_SE
BD-PH	Predicted 66	SusC ▶ SusD ▶ unk ▶
BD-PH	Predicted 67	SusC ▶ SusD ▶
BD-PH	Predicted 68	SusC ▶ SusD ▶
BD-PH	Predicted 69	SusC ▶ SusD ▶ GH29 ▶ SusC ▶ SusD ▶
BD-PH	Predicted 70	SusC ▶ SusD ▶ unk ▶ GH123 ▶ GH109 ▶ GH36 ▶ unk ▶ unk ▶ unk ▶ unk ▶ unk ▶ unk ▶ Sulf_1 ▶ unk ▶ ◀ unk GH36 ▶ unk ▶ unk ▶ unk ▶
BD-PH	Predicted 71	Anti-σ ▶ unk ▶ SusC ▶ SusC ▶ SusD ▶
BD-PH	Predicted 72	HTCS ▶ GH31 ▶ GH2 ▶ SusC ▶ SusD ▶ unk ▶ unk ▶ GH5_4 ▶
BD-PH	Predicted 73	unk ▶ SusC ▶ SusD ▶ unk ▶
BD-PH	Predicted 74	SusC ▶ SusD ▶ unk ▶ unk ▶ GH116|CBM38 ▶ unk ▶ unk ▶
BD-PH	Predicted 75	◀ unk ◀ unk ◀ GH105 unk ▶ unk ▶ SusC ▶ SusD ▶ GH95 ▶ SusC ▶ SusC ▶ SusD ▶ unk ▶ CE8|PL10 ▶ GH2 ▶ CE7 ▶ GH28 ▶ CE12 ▶ GH43 ▶ CE8 ▶ HTCS ▶
BD-PH	Predicted 76	GH29 ▶ ◀ GH2 ◀ unk SusC ▶ SusD ▶ SusC ▶ SusD ▶ unk ▶ unk ▶ GH16 ▶ HTCS ▶ GH16 ▶ GH123 ▶ Pept_SC ▶ GH2 ▶ GH42 ▶
BD-PH	Predicted 77	unk ▶ GH92 ▶ SusC ▶ SusD ▶ unk ▶
BD-PH	Predicted 78	SusC ▶ SusD ▶ unk ▶ unk ▶
BD-PH	Predicted 79	SusC ▶ SusD ▶
BD-PH	Predicted 80	SusC ▶ SusD ▶ GH2 ▶ GH43 ▶ unk ▶ GH35 ▶ HTCS ▶
BD-PH	Predicted 81	◀ HTCS SusC ▶ SusD ▶
BD-PH	Predicted 82	◀ ECF-σ Anti-σ ▶ SusC ▶ SusD ▶ unk ▶ unk ▶
BD-PH	Predicted 83	SusR ▶ SusC ▶ SusD ▶
BD-PH	Predicted 84	SusC ▶ SusD ▶
BD-PH	Predicted 85	◀ GH2 ◀ GH28 ◀ unk ◀ SusD ◀ SusC HTCS ▶ ◀ unk SusC ▶ SusD ▶ unk ▶ unk ▶
BD-PH	Predicted 86	SusC ▶ SusD ▶ unk ▶ unk ▶
BD-PH	Predicted 87	unk ▶ unk ▶ Anti-σ ▶ SusC ▶ SusD ▶ unk ▶ GH2 ▶ unk ▶
BD-PH	Predicted 88	ECF-σ ▶ ◀ unk Anti-σ ▶ SusC ▶ SusD ▶ GH92 ▶ CE15 ▶ GH92 ▶

BD-PH	Predicted 89	SusC ▶ SusD ▶ unk ▶ GH13 ▶ GH13 ▶
BD-PH	Predicted 90	ECF-σ ▶ Anti-σ ▶ SusC ▶ SusD ▶ unk ▶ GH30_3 ▶ unk ▶
BD-PH	Predicted 91	ECF-σ ▶ ◀ unk Anti-σ ▶ SusC ▶ SusD ▶ GH127 ▶ GH127 ▶ unk ▶ unk ▶
BD-PH	Predicted 92	CBM38 ▶ MFS ▶ unk ▶ SusC ▶ SusD ▶ unk ▶ CE1 ▶ GH13 ▶ SusC|CE1|CBM48 ▶
BD-PH	Predicted 93	SusC ▶ SusD ▶
BD-PH	Predicted 94	◀ unk ◀ GH130 SusC ▶ SusD ▶ unk ▶ unk ▶ unk ▶ unk ▶ unk ▶ unk ▶ unk ▶
BD-PH	Predicted 95	SusC ▶ SusD ▶ Pept_MC ▶
BD-PH	Predicted 96	CBM6|GH128 ▶ unk ▶ CBM6|GH81|CBM38 ▶ GH3|CBM32 ▶ CBM6|GH5_46 ▶ unk ▶ SusC ▶ SusD ▶ GH16 ▶ CBM6|GH128 ▶ GH2 ▶ GH16 ▶
BD-PH	Predicted 97	HTCS ▶ ◀ unk GH95 ▶ GH98|CBM35 ▶ SusC ▶ SusD ▶ SusC ▶ SusD ▶ unk ▶ GH10|CBM4|GH10 ▶ unk ▶ unk ▶ unk ▶ unk ▶ unk ▶ GH115|GH10 ▶ GH43 ▶ unk ▶ GH29 ▶ GH43 ▶ unk ▶ unk ▶ GH108 ▶
BD-PH	Predicted 98	SusC ▶ SusD ▶ CBM48 ▶
BD-PH	Predicted 99	unk ▶ SusC ▶ SusC ▶ SusD ▶ SusD ▶
BD-PH	Predicted 100	SusC ▶ SusD ▶
BD-PH	Predicted 101	SusC ▶ SusD ▶ unk ▶ unk ▶
BD-PH	Predicted 102	ECF-σ ▶ Anti-σ ▶ SusC ▶ SusD ▶ unk ▶ GH5_4 ▶ GH31 ▶ GH31 ▶ unk ▶ GH29 ▶ ◀ GH2 SusC ▶ SusD ▶ unk ▶ GH10|CBM4 ▶ GH35 ▶ GH115 ▶ GH67 ▶ unk ▶ MFS ▶ GH10 ▶ GH11 ▶ GH43 ▶ GH3 ▶ unk ▶
BD-PH	Predicted 103	GH3 ▶ unk ▶ SusC ▶ SusD ▶ unk ▶ unk ▶
BD-PH	Predicted 104	SusR ▶ ◀ GH87 unk ▶ SusC ▶ SusD ▶ GH97 ▶
BD-PH	Predicted 105	SusC ▶ SusD ▶ unk ▶ GH16 ▶ GH3 ▶
BD-PH	Predicted 106	SusC ▶ SusD ▶
BD-PH	Predicted 107	SusC ▶ SusD ▶ unk ▶
BD-PH	Predicted 108	GH3 ▶ SusC ▶ SusD ▶ unk ▶ GH30_3 ▶ GH30_3 ▶
BD-PH	Predicted 109	SusC ▶ SusD ▶ unk ▶ GH10|CBM4 ▶ GH35 ▶ unk ▶ MFS ▶ GH10 ▶ GH43 ▶ GH67 ▶ GH43 ▶
BD-PH	Predicted 110	SusC ▶ SusD ▶ unk ▶

BD-PH	Predicted 111	SusC ▶ SusD ▶ GH106 ▶
BD-PH	Predicted 112	◀ SusC SusC ▶ SusD ▶
BD-PH	Predicted 113	SusC ▶ SusD ▶
BD-PH	Predicted 114	ECF-σ ▶ Anti-σ ▶ SusC ▶ SusD ▶
BD-PH	Predicted 115	SusC ▶ SusD ▶ GH105 ▶
BD-PH	Predicted 116	SusR ▶ SusC ▶ SusD ▶ unk ▶ GH16 ▶ GH3 ▶ unk ▶
BD-PH	Predicted 117	unk ▶ SusC ▶ SusD ▶ unk ▶ GH2 ▶ GH2 ▶ GH43 ▶ unk ▶ unk ▶ GH115 ▶ CBM66|GH32 ▶ unk ▶ CBM66|GH51 ▶ GH31 ▶ GH43 ▶ CBM62 ▶ GH78 ▶ CBM32 ▶ GH92 ▶ unk ▶ CBM13|GH76|CBM6 ▶ CBM66 ▶
BD-PH	Predicted 118	GH127 ▶ SusC ▶ SusD ▶ unk ▶
BD-PH	Predicted 119	SusC ▶ SusD ▶ Pept_SC ▶
BD-PH	Predicted 120	SusC ▶ SusD ▶ ◀ unk ◀ unk GH106 ▶
BD-PH	Predicted 121	unk ▶ SusC ▶ SusD ▶
BD-PH	Predicted 122	SusC ▶ SusD ▶ unk ▶ unk ▶ GH2 ▶ unk ▶ unk ▶ unk ▶ GH92 ▶ unk ▶ unk ▶ unk ▶ MFS ▶ GH92 ▶
BD-PH	Predicted 123	AraC ▶ GH78 ▶ GH78 ▶ Sulf_4|GH20 ▶ unk ▶ SusC ▶ SusD ▶ SusC ▶ SusD ▶
BD-PH	Predicted 124	SusC ▶ SusD ▶ unk ▶ CBM6 ▶ unk ▶ unk ▶ GH127 ▶ GH78 ▶ SusC ▶ SusD ▶ unk ▶ unk ▶
BD-PH	Predicted 125	SusC ▶ SusD ▶ GH33 ▶ unk ▶ GH33 ▶ GH33 ▶ MFS ▶
BD-PH	Predicted 126	ECF-σ ▶ Anti-σ ▶ SusC ▶ SusD ▶ GH105 ▶ ◀ GH78 ◀ GH78 ◀ GH78 ◀ GH78 GntR ▶
BD-PH	Predicted 127	GH105 ▶ unk ▶ ◀ GH2 ◀ HTCS SusC ▶ SusD ▶ GH43|GH16 ▶ ◀ unk ◀ unk GH2 ▶ HTCS ▶ ◀ GH43 ◀ SusD ◀ SusC ◀ unk ◀ unk GH43 ▶ ◀ unk GH97 ▶ GH51 ▶ unk ▶ unk ▶ unk ▶ unk ▶ GH51 ▶ GH43 ▶ GH43 ▶ GH35|CBM32 ▶ GH51 ▶
BD-PH	Predicted 128	ECF-σ ▶ Anti-σ ▶ SusC ▶ unk ▶ unk ▶ SusC ▶ unk ▶ SusC ▶ SusD ▶
BD-PH	Predicted 129	SusC ▶ SusD ▶ unk ▶
BD-PH	Predicted 130	SusC ▶ unk ▶ SusD ▶ unk ▶
BD-PH	Predicted 131	SusR ▶ SusC ▶ SusD ▶ unk ▶ GH27 ▶ GH97 ▶

BD-PH	Predicted 132	◀ GH3 ◀ unk CE1 ▶ CE1 ▶ ◀ HTCS SusC ▶ SusD ▶ GH43 ▶ CE6|CE1 ▶ GH9 ▶ GH43|CBM32|CBM48|CE1 ▶ CBM48|CE1 ▶ GH51 ▶ CE1 ▶ CE1 ▶ unk ▶ GH43|CBM6 ▶ unk ▶
BD-PH	Predicted 133	SusC ▶ SusD ▶ unk ▶ unk ▶ GH9 ▶ MFS ▶ GH10 ▶ GH3 ▶ ◀ CE1 ◀ CE1 ◀ GH3 ◀ GH51
BD-PH	Predicted 134	SusC ▶ SusD ▶
BD-PH	Predicted 135	MFS ▶ SusC ▶ SusD ▶ unk ▶ unk ▶ GH13|CBM58 ▶ unk ▶ GH13 ▶ GH13 ▶ unk ▶ unk ▶ unk ▶ unk ▶
BD-PH	Predicted 136	◀ HTCS GH2 ▶ unk ▶ SusC ▶ SusD ▶ PL9 ▶
BD-PH	Predicted 137	unk ▶ SusC ▶ SusD ▶ CE1 ▶ GH3 ▶ GH51 ▶ MFS ▶ unk ▶ unk ▶
BD-PH	Predicted 138	SusC ▶ SusD ▶ unk ▶ ◀ GH2 ◀ GH53|GH53 ◀ unk
BD-PH	Predicted 139	SusC ▶ SusD ▶ unk ▶ ◀ GH2 ◀ GH53|GH53 ◀ GH2 ◀ HTCS GH3 ▶ unk ▶ ◀ unk GH51 ▶
BD-PH	Predicted 140	◀ GH3 ◀ unk CE1 ▶ CE1 ▶ ◀ HTCS SusC ▶ SusD ▶ GH43 ▶ CE1|CE6 ▶ GH9 ▶ CE1|CBM48|CBM32|GH43 ▶ CE1|CBM48 ▶ GH51 ▶ CE1 ▶ CE1 ▶ unk ▶ CBM6|GH43 ▶ unk ▶
BD-PH	Predicted 141	ECF-σ ▶ Anti-σ ▶ SusC ▶ SusD ▶ Pept_SC ▶ GH31 ▶ unk ▶
BD-PH	Predicted 142	SusC ▶ SusD ▶ CBM32|GH43 ▶ Anti-σ ▶ SusC ▶
BD-PH	Predicted 143	SusC ▶ SusD ▶ unk ▶ ◀ GH2 ◀ GH53|GH53 ◀ GH2 ◀ HTCS GH3 ▶ unk ▶ ◀ unk GH51 ▶
BD-PH	Predicted 144	HTCS ▶ unk ▶ unk ▶ GH127 ▶ unk ▶ SusC ▶ SusD ▶ unk ▶ unk ▶
BD-PH	Predicted 145	SusR ▶ SusC ▶ SusD ▶ unk ▶ GH16 ▶ GH3 ▶ unk ▶
BD-PH	Predicted 146	ECF-σ ▶ Anti-σ ▶ SusC ▶ SusD ▶ Pept_SC ▶ GH31 ▶ unk ▶
BD-PH	Predicted 147	SusR ▶ SusC ▶ SusD ▶ unk ▶ GH66 ▶ GH31 ▶ CBM35 ▶
BD-PH	Predicted 148	ECF-σ ▶ Anti-σ ▶ SusC ▶ SusD ▶ Pept_SC ▶ GH31 ▶ unk ▶
BD-PH	Predicted 149	GH127 ▶ SusC ▶ SusD ▶ unk ▶
BD-PH	Predicted 150	◀ HTCS GH2 ▶ unk ▶ SusC ▶ SusD ▶ PL9 ▶
BD-PH	Predicted 151	GH127 ▶ SusC ▶ SusD ▶ unk ▶
BD-PH	Predicted 152	GH127 ▶ SusC ▶ SusD ▶ unk ▶
BD-PH	Predicted 153	GH43 ▶ ◀ unk ◀ GH130 SusC ▶ SusD ▶ unk ▶ unk ▶ unk ▶ unk ▶ unk ▶ unk ▶ unk ▶

BD-PH	Predicted 154	GH43 ▶ ◀ unk ◀ GH130 SusC ▶ SusD ▶ unk ▶ unk ▶ unk ▶ unk ▶ unk ▶ unk ▶ unk ▶
BD-PH	Predicted 155	MFS ▶ SusC ▶ SusD ▶ unk ▶ unk ▶ CBM58|GH13 ▶ unk ▶ GH13 ▶ GH13 ▶ unk ▶ unk ▶ unk ▶ unk ▶
BD-PH	Predicted 156	ECF-σ ▶ Anti-σ ▶ unk ▶ SusC ▶ SusC ▶ unk ▶ SusD ▶
BD-PH	Predicted 157	◀ unk ◀ GH130 SusC ▶ SusD ▶ unk ▶ unk ▶ unk ▶ unk ▶ unk ▶ unk ▶ unk ▶
BD-PH	Predicted 158	unk ▶ unk ▶ unk ▶ GH36 ▶ SusC ▶ SusD ▶ unk ▶ unk ▶ unk ▶ unk ▶ unk ▶ ◀ CE4
BD-PH	Predicted 159	SusC ▶ SusD ▶ GH2 ▶ GH43 ▶ unk ▶ GH35 ▶ HTCS ▶
BD-PH	Predicted 160	◀ HTCS SusC ▶ SusD ▶ Pept_PB ▶ ◀ HTCS CBM38 ▶ MFS ▶ unk ▶ SusC ▶ SusD ▶ unk ▶ CE1 ▶ GH13 ▶ SusC CBM48|CE1 ▶
BD-PH	Predicted 161	◀ HTCS SusC ▶ SusD ▶
BD-PH	Predicted 162	SusC ▶ SusD ▶ GH2 ▶ GH43 ▶ unk ▶ GH35 ▶ HTCS ▶
BD-PH	Predicted 163	SusR ▶ SusC ▶ SusD ▶ unk ▶ GH27 ▶ GH97 ▶
BD-PH	Predicted 164	SusR ▶ SusC ▶ SusD ▶
BD-PH	Predicted 165	SusR ▶ SusC ▶ SusD ▶ unk ▶ GH16 ▶ GH3 ▶ unk ▶
BD-PH	Predicted 166	SusC ▶ SusD ▶ GH43 ▶ Sulf_1 ▶ GH31 ▶ HTCS ▶ GH2 ▶ GH93 ▶ GH43 ▶ GH5_13 ▶ GH38 ▶
BD-PH	Predicted 167	SusR ▶ SusC ▶ SusD ▶ unk ▶ GH27 ▶ GH97 ▶
BD-PH	Predicted 168	SusR ▶ SusC ▶ SusD ▶ unk ▶ GH27 ▶ GH97 ▶
BD-PH	Predicted 169	SusC ▶ SusD ▶ GH78 ▶
BD-PH	Predicted 170	SusC ▶ SusC ▶ SusD ▶ unk ▶ unk ▶ unk ▶ ◀ unk SusC ▶ SusD ▶
BD-PH	Predicted 171	SusC ▶ SusD ▶
MR-C	Predicted 1	unk ▶ MFS ▶ unk ▶ SusC ▶ SusD ▶ unk ▶ unk ▶ Pept_PB ▶ ◀ CBM20 |GH77
MR-C	Predicted 2	ECF-σ ▶ Anti-σ ▶ SusC ▶ SusD ▶ unk ▶ unk ▶
MR-C	Predicted 3	SusC ▶ SusD ▶ unk ▶
MR-C	Predicted 4	ECF-σ ▶ Anti-σ ▶ SusC ▶ SusD ▶ Pept_MC ▶
MR-C	Predicted 5	SusC ▶ SusD ▶ unk ▶ unk ▶ unk ▶ unk ▶

MR-C	Predicted 6	SusC ▶ SusD ▶ unk ▶ GH28 ▶ CE12 ▶ GH105 ▶ CE8 ▶ PL1 ▶ PL1 ▶ CE8 ▶ unk ▶ PL1|CBM77 ▶ CE8 ▶ unk ▶
MR-C	Predicted 7	ECF-σ ▶ Anti-σ ▶ SusC ▶ SusD ▶ unk ▶ unk ▶ Pept_PC ▶ unk ▶ Pept_MC ▶ unk ▶ unk ▶ Pept_na ▶ Pept_na ▶
MR-C	Predicted 8	unk ▶ SusC ▶ SusC ▶ SusD ▶ unk ▶ unk ▶
MR-C	Predicted 9	SusC ▶ SusD ▶ GH43_3 ▶ GH31 ▶ unk ▶ unk ▶ unk ▶ GH43_31 ▶ Sulf_1 ▶ GH31 ▶ HTCS ▶ GH2 ▶ GH93 ▶ GH43_3 ▶ GH5_13 ▶ GH38 ▶
MR-C	Predicted 10	SusR ▶ SusC ▶ SusD ▶ unk ▶ unk ▶ GH27 ▶ GH66 ▶ GH31 ▶ CBM35 ▶
MR-C	Predicted 11	GH3 ▶ unk ▶ SusC ▶ SusD ▶ unk ▶ unk ▶
MR-C	Predicted 12	ECF-σ ▶ Anti-σ ▶ SusC ▶ SusD ▶ unk ▶ unk ▶ unk ▶ GH92 ▶ MFS ▶ unk ▶ unk ▶
MR-C	Predicted 13	SusR ▶ ECF-σ ▶ unk ▶ GH38 ▶ GH92 ▶ GH78|CBM67 ▶ GH92 ▶ GH92 ▶ GH130 ▶ SusD ▶ unk ▶ unk ▶ SusC ▶ SusD ▶ SusC ▶
MR-C	Predicted 14	SusC ▶ SusD ▶
MR-C	Predicted 15	ECF-σ ▶ Anti-σ ▶ SusC ▶ SusD ▶ unk ▶
MR-C	Predicted 16	SusC ▶ SusD ▶
MR-C	Predicted 17	SusC ▶ SusD ▶
MR-C	Predicted 18	ECF-σ ▶ Anti-σ ▶ SusC ▶ SusC ▶ SusD ▶ unk ▶
MR-C	Predicted 19	SusC ▶ SusD ▶ unk ▶ unk ▶ GH26 ▶ GH26 ▶ GH130 ▶ GH27 ▶ GH5_25 ▶ MFS ▶ unk ▶ GH5_2 ▶ unk ▶ GH3 ▶
MR-C	Predicted 20	ECF-σ ▶ Anti-σ ▶ SusC ▶ SusD ▶ GH20 ▶ unk ▶ GH20 ▶
MR-C	Predicted 21	ECF-σ ▶ SusC ▶ SusD ▶ unk ▶ Anti-σ ▶
MR-C	Predicted 22	unk ▶ unk ▶ SusC ▶ SusD ▶ unk ▶ GH2 ▶ GH2 ▶ GH43_3 ▶ unk ▶ unk ▶ GH115 ▶ GH32|CBM66 ▶ unk ▶ GH51|CBM66 ▶ GH31 ▶ GH43_31 ▶ CBM62 ▶ GH78|CBM67 ▶ CBM32 ▶ GH92 ▶ unk ▶ CBM6|GH76|CBM13 ▶ CBM66 ▶
MR-C	Predicted 23	SusC ▶ SusD ▶ unk ▶
MR-C	Predicted 24	GH128|CBM6 ▶ unk ▶ CBM38|GH81|CBM6 ▶ CBM32|GH3 ▶ GH5_46|CBM6 ▶ unk ▶ SusC ▶ SusD ▶ GH16 ▶ GH128|CBM6 ▶ unk ▶ GH2 ▶ GH16 ▶
MR-C	Predicted 25	SusC ▶ SusD ▶

<table>
<tr><td>MR-C</td><td>Predicted 26</td><td>◀ HTCS PL1 ▶ SusC ▶ SusD ▶ unk ▶ GH28 ▶ unk ▶ CE8 ▶ GH95 ▶ CE12|GH43_10 ▶</td></tr>
<tr><td>MR-C</td><td>Predicted 27</td><td>ECF-σ ▶ Anti-σ ▶ SusC ▶ unk ▶ SusD ▶ unk ▶ GH5_4 ▶ GH31 ▶ GH31 ▶ GH29 ▶ ◀ GH2 SusC ▶ SusD ▶ unk ▶ GH10|CBM4 ▶ SusC ▶ SusD ▶ GH35 ▶ GH115 ▶ GH67 ▶ unk ▶ MFS ▶ GH10 ▶ GH11 ▶ GH43_1 ▶ GH3 ▶ unk ▶</td></tr>
<tr><td>MR-C</td><td>Predicted 28</td><td>GH3 ▶ SusC ▶ SusD ▶ unk ▶ GH30_3 ▶ GH30_3 ▶</td></tr>
<tr><td>MR-C</td><td>Predicted 29</td><td>GntR ▶ SusC ▶ SusD ▶ unk ▶ PL6 ▶ GH88 ▶ unk ▶ unk ▶ PL12 ▶ unk ▶ unk ▶ unk ▶ PL6 ▶ PL17 ▶ unk ▶ MFS ▶ unk ▶</td></tr>
<tr><td>MR-C</td><td>Predicted 30</td><td>SusR ▶ ◀ GH87 unk ▶ SusC ▶ SusD ▶ GH97 ▶</td></tr>
<tr><td>MR-C</td><td>Predicted 31</td><td>SusC ▶ SusD ▶ unk ▶ GH16 ▶ GH3 ▶</td></tr>
<tr><td>MR-C</td><td>Predicted 32</td><td>unk ▶ GH76 ▶ SusC ▶ SusD ▶ unk ▶ unk ▶ unk ▶ GH92 ▶ GH125 ▶</td></tr>
<tr><td>MR-PH</td><td>Predicted 1</td><td>SusC ▶ SusD ▶ unk ▶</td></tr>
<tr><td>MR-PH</td><td>Predicted 2</td><td>ECF-σ ▶ Anti-σ ▶ SusC ▶ SusD ▶ Pept_MC ▶</td></tr>
<tr><td>MR-PH</td><td>Predicted 3</td><td>SusC ▶ SusD ▶ unk ▶</td></tr>
<tr><td>MR-PH</td><td>Predicted 4</td><td>◀ GH3 ◀ unk CE1 ▶ CE1 ▶ ◀ HTCS SusC ▶ SusD ▶ GH43_17 ▶ CE1|CE6 ▶ GH9 ▶ CE1|CBM48|CBM32|GH43_28 ▶ CE1|CBM48 ▶ GH51 ▶ CE1 ▶ CE1 ▶ unk ▶ CBM6|GH43_2 ▶ unk ▶ CE15 ▶</td></tr>
<tr><td>MR-PH</td><td>Predicted 5</td><td>SusR ▶ SusC ▶ SusD ▶ unk ▶ GH27 ▶ GH97 ▶</td></tr>
<tr><td>MR-PH</td><td>Predicted 6</td><td>unk ▶ unk ▶ unk ▶ GH36 ▶ SusC ▶ SusD ▶ unk ▶ unk ▶ unk ▶ unk ▶ unk ▶ ◀ CE4</td></tr>
<tr><td>MR-PH</td><td>Predicted 7</td><td>◀ GH95 ◀ unk ◀ GH67|GH43_10 ◀ HTCS MFS ▶ GH43_1 ▶ ◀ unk ◀ GH130 SusC ▶ SusD ▶ unk ▶ unk ▶ unk ▶ unk ▶ unk ▶ unk ▶ unk ▶</td></tr>
<tr><td>MR-PH</td><td>Predicted 8</td><td>◀ GH3 ◀ unk CE1 ▶ CE1 ▶ ◀ HTCS SusC ▶ SusD ▶ GH43_17 ▶ CE6|CE1 ▶ GH9 ▶ GH43_28|CBM32|CBM48|CE1 ▶ CBM48|CE1 ▶ GH51 ▶ CE1 ▶ CE1 ▶ unk ▶ GH43_2|CBM6 ▶ unk ▶ CE15 ▶</td></tr>
<tr><td>MR-PH</td><td>Predicted 9</td><td>SusC ▶ SusD ▶ GH2 ▶ GH43_24 ▶ unk ▶ GH35 ▶ HTCS ▶</td></tr>
<tr><td>MR-PH</td><td>Predicted 10</td><td>◀ HTCS GH32|CBM38 ▶ MFS ▶ unk ▶ unk ▶ SusC ▶ SusD ▶ unk ▶ GH13_18 ▶ MFS ▶ unk ▶ unk ▶</td></tr>
<tr><td>MR-PH</td><td>Predicted 11</td><td>ECF-σ ▶ SusC ▶ SusD ▶ unk ▶ Anti-σ ▶</td></tr>
<tr><td>MR-PH</td><td>Predicted 12</td><td>ECF-σ ▶ Anti-σ ▶ SusC ▶ SusD ▶ unk ▶ unk ▶ Pept_PC ▶ unk ▶ Pept_MC ▶ unk ▶ unk ▶ Pept_na ▶ Pept_na ▶</td></tr>
</table>

MR-PH	Predicted 13	SusC ▶ SusD ▶ unk ▶ ◀ GH2 ◀ GH53|GH53 ◀ GH2
MR-PH	Predicted 14	SusC ▶ SusD ▶
MR-PH	Predicted 15	ECF-σ ▶ Anti-σ ▶ SusC ▶ SusD ▶ unk ▶ unk ▶ unk ▶
MR-PH	Predicted 16	unk ▶ SusC ▶ SusD ▶
MR-PH	Predicted 17	unk ▶ MFS ▶ unk ▶ SusC ▶ SusD ▶ unk ▶ unk ▶ Pept_PB ▶ ◀ GH77 |CBM20
MR-PH	Predicted 18	SusC ▶ SusD ▶
MR-PH	Predicted 19	SusC ▶ SusD ▶
MR-PH	Predicted 20	SusC ▶ SusD ▶
MR-PH	Predicted 21	SusC ▶ SusD ▶
MR-PH	Predicted 22	SusC ▶ SusD ▶ unk ▶
MR-PH	Predicted 23	unk ▶ SusC ▶ SusD ▶ CE1 ▶ GH3 ▶ GH51 ▶ MFS ▶ unk ▶ unk ▶
MR-PH	Predicted 24	◀ GH3 ◀ unk CE1 ▶ CE1 ▶ ◀ HTCS SusC ▶ SusD ▶ GH43_17 ▶ CE1| CE6 ▶ GH9 ▶ CE1|CBM48|CBM32|GH43_28 ▶ CE1|CBM48 ▶ GH51 ▶ CE1 ▶ CE1 ▶ unk ▶ CBM6|GH43_2 ▶ unk ▶ CE15 ▶
MR-PH	Predicted 25	SusC ▶ SusD ▶ unk ▶
MR-PH	Predicted 26	ECF-σ ▶ Anti-σ ▶ SusC ▶ SusD ▶ unk ▶
MR-PH	Predicted 27	SusC ▶ SusD ▶
MR-PH	Predicted 28	SusC ▶ SusD ▶ unk ▶
MR-PH	Predicted 29	SusC ▶ SusD ▶ unk ▶ unk ▶ GH50|GH43_17 ▶
MR-PH	Predicted 30	GH127 ▶ SusC ▶ SusD ▶ unk ▶
MR-PH	Predicted 31	SusC ▶ SusD ▶
MR-PH	Predicted 32	SusC ▶ SusD ▶
MR-PH	Predicted 33	SusC ▶ SusD ▶
MR-PH	Predicted 34	ECF-σ ▶ Anti-σ ▶ SusC ▶ SusD ▶ Pept_SC ▶ GH31 ▶ unk ▶
MR-PH	Predicted 35	SusC ▶ SusD ▶ unk ▶
MR-PH	Predicted 36	SusC ▶ SusD ▶ unk ▶
MR-PH	Predicted 37	SusC ▶ SusD ▶ unk ▶
MR-PH	Predicted 38	SusC ▶ SusD ▶ unk ▶

MR-PH	Predicted 39	MFS ▶ SusC ▶ SusD ▶ unk ▶ unk ▶ CBM58|GH13 ▶ unk ▶ GH13 ▶ GH13 ▶ unk ▶ unk ▶ unk ▶ unk ▶
MR-PH	Predicted 40	ECF-σ ▶ Anti-σ ▶ SusC ▶ SusD ▶ Pept_SC ▶ GH31 ▶ unk ▶
MR-PH	Predicted 41	ECF-σ ▶ Anti-σ ▶ SusC ▶ SusD ▶ Pept_SC ▶ GH31 ▶ unk ▶
MR-PH	Predicted 42	Pept_SC ▶ unk ▶ SusC ▶ SusD ▶
MR-PH	Predicted 43	SusC ▶ SusD ▶ unk ▶ GH16 ▶ GH3 ▶ unk ▶ unk ▶ ◀ Sulf_4
MR-PH	Predicted 44	SusC ▶ SusD ▶ unk ▶ GH16 ▶ GH3 ▶ unk ▶ unk ▶ ◀ Sulf_4
MR-PH	Predicted 45	Pept_SC ▶ unk ▶ SusC ▶ SusD ▶
MR-PH	Predicted 46	SusR ▶ SusC ▶ SusD ▶ unk ▶ GH16 ▶ GH3 ▶ unk ▶ unk ▶ ◀ Sulf_4
MR-PH	Predicted 47	Pept_SC ▶ unk ▶ SusC ▶ SusD ▶
MR-PH	Predicted 48	unk ▶ SusC ▶ SusD ▶ unk ▶ unk ▶
MR-PH	Predicted 49	ECF-σ ▶ Anti-σ ▶ SusC ▶ SusD ▶ Pept_SC ▶ GH31 ▶ unk ▶
MR-PH	Predicted 50	◀ HTCS CBM38 ▶ MFS ▶ unk ▶ SusC ▶ SusD ▶ unk ▶ CE1 ▶ GH13 ▶ SusC|CBM48|CE1 ▶
MR-PH	Predicted 51	◀ SusC SusC ▶ SusD ▶ unk ▶ unk ▶
MR-PH	Predicted 52	SusR ▶ SusC ▶ SusD ▶ unk ▶
MR-PH	Predicted 53	SusR ▶ SusC ▶ SusD ▶ unk ▶
MR-PH	Predicted 54	unk ▶ GH127 ▶ unk ▶ SusC ▶ SusD ▶ unk ▶ unk ▶
MR-PH	Predicted 55	unk ▶ unk ▶ GH127 ▶ unk ▶ SusC ▶ SusD ▶ unk ▶ unk ▶
MR-PH	Predicted 56	MFS ▶ SusC ▶ SusD ▶ unk ▶ unk ▶ CBM58|GH13 ▶ unk ▶ GH13 ▶ GH13 ▶ unk ▶ unk ▶ unk ▶ unk ▶
MR-PH	Predicted 57	MFS ▶ SusC ▶ SusD ▶ unk ▶ unk ▶ CBM58|GH13 ▶ unk ▶ GH13 ▶ GH13 ▶ unk ▶ unk ▶ unk ▶ unk ▶
MR-PH	Predicted 58	MFS ▶ SusC ▶ SusD ▶ unk ▶ unk ▶ GH13|CBM58 ▶ unk ▶ GH13 ▶ GH13 ▶ unk ▶ unk ▶ unk ▶ unk ▶
MR-PH	Predicted 59	ECF-σ ▶ Anti-σ ▶ SusC ▶ SusD ▶ unk ▶ unk ▶
MR-PH	Predicted 60	SusC ▶ SusD ▶ unk ▶ unk ▶ SusC ▶ SusD ▶
MR-PH	Predicted 61	SusC ▶ SusD ▶
MR-PH	Predicted 62	SusC ▶ SusD ▶
MR-PH	Predicted 63	Pept_SC ▶ unk ▶ SusC ▶ SusD ▶

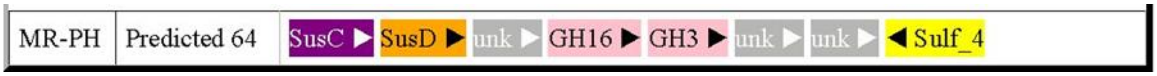

Figure S4 Predicted PULs from the metagenomes. BD-C, beaver-droppings enriched on cellulose; BD-PH, beaver-droppings enriched on poplar hydrolysate; MR-C, moose rumen enriched on cellulose; MR-PH, moose rumen enriched on poplar hydrolysate.

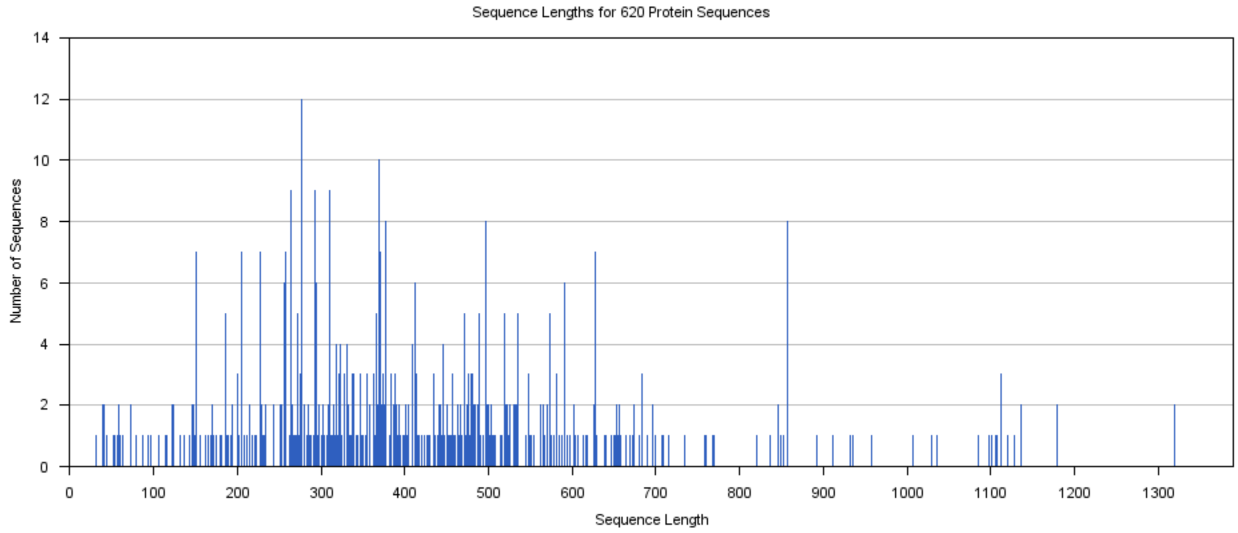

Figure S5a Distribution of sequence length for proteins of unknown function positioned within predicted PULs.

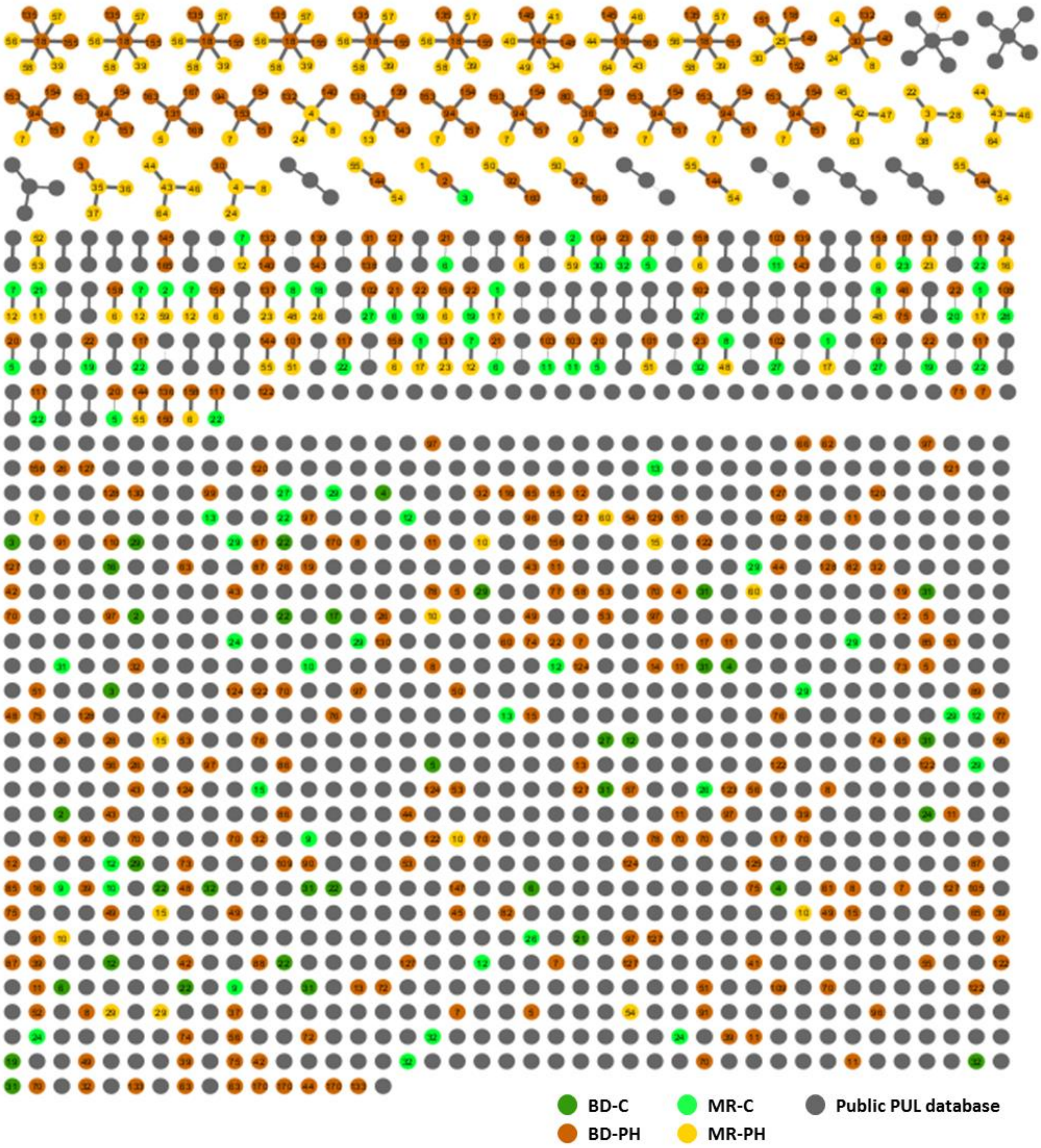

Figure S5b Similarity-based clustering (≥70%) of proteins of unknown function positioned within PULs predicted herein and the public PUL database (http://www.cazy.org/PULDB/).

Each dot represents a protein of unknown function with its PUL identifier shown on top; the thickness of the edges correlates with percent identity.

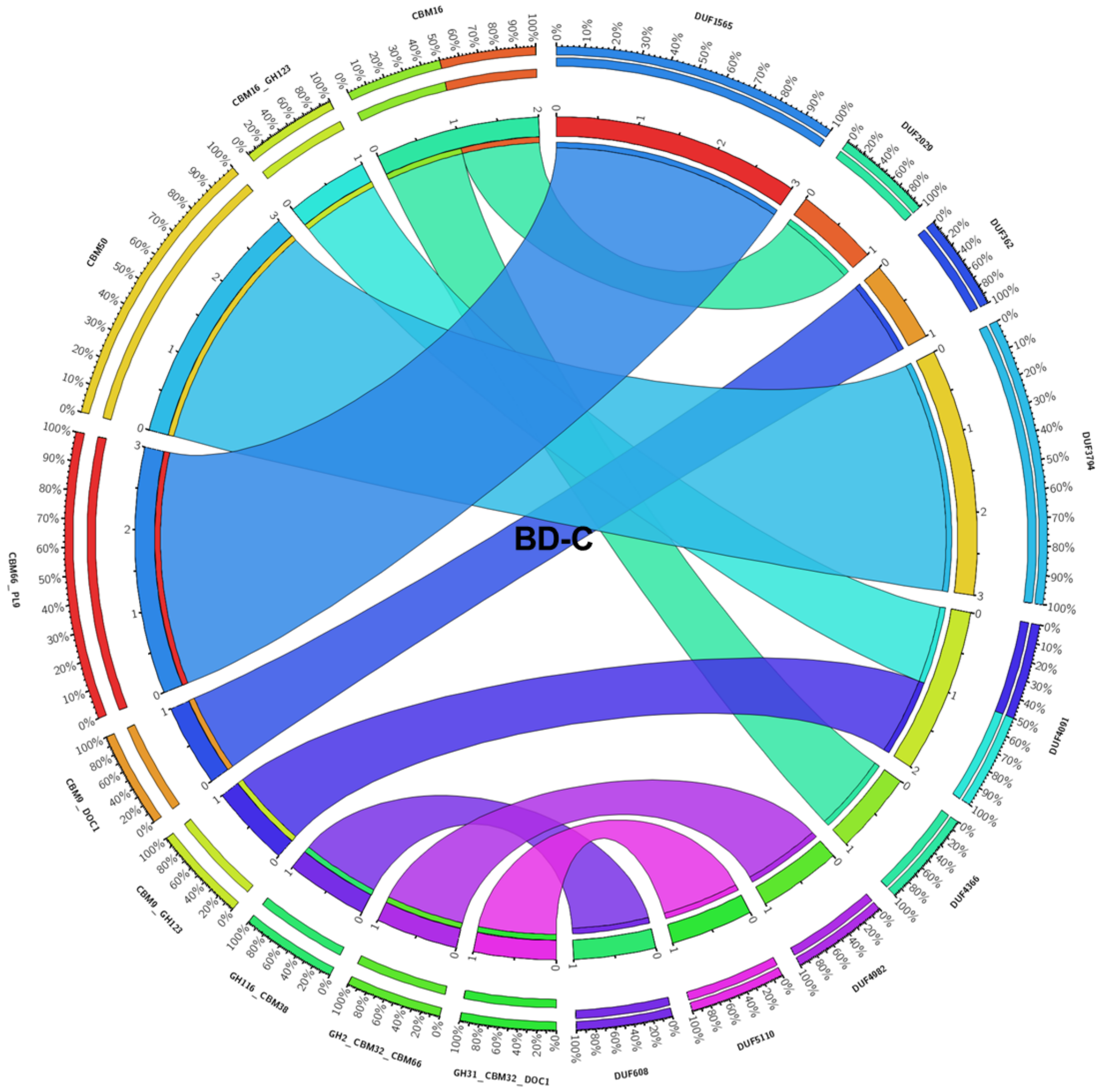
BD-C
CBM16
DUF1565
DUF2020
DUF362
DUF3794
DUF4091
DUF4366
DUF4982
DUF5110
DUF608
GH31_CBM32_DOC1
GH2_CBM32_CBM66
GH116_CBM38
CBM0_GH123
CBM0_DOC1
CBM66_PL9
CBM50
CBM16_GH123

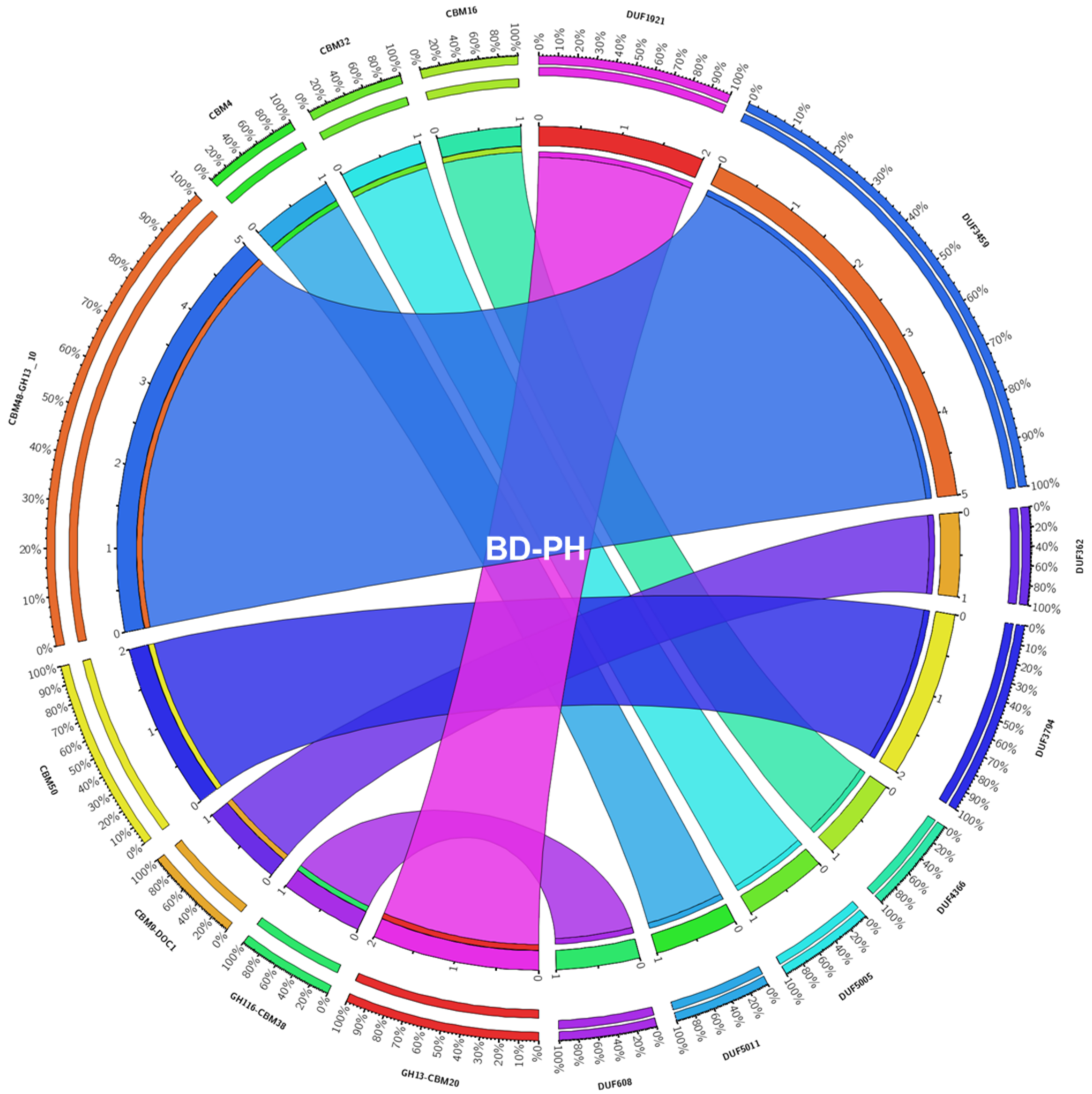
BD-PH
CBM16
DUF1921
CBM32
CBM4
DUF3459
CBM48-GH13_10
DUF362
DUF3704
CBM50
DUF4366
CBM9-DOC1
DUF5005
GH116-CBM38
DUF5011
GH13-CBM20
DUF608

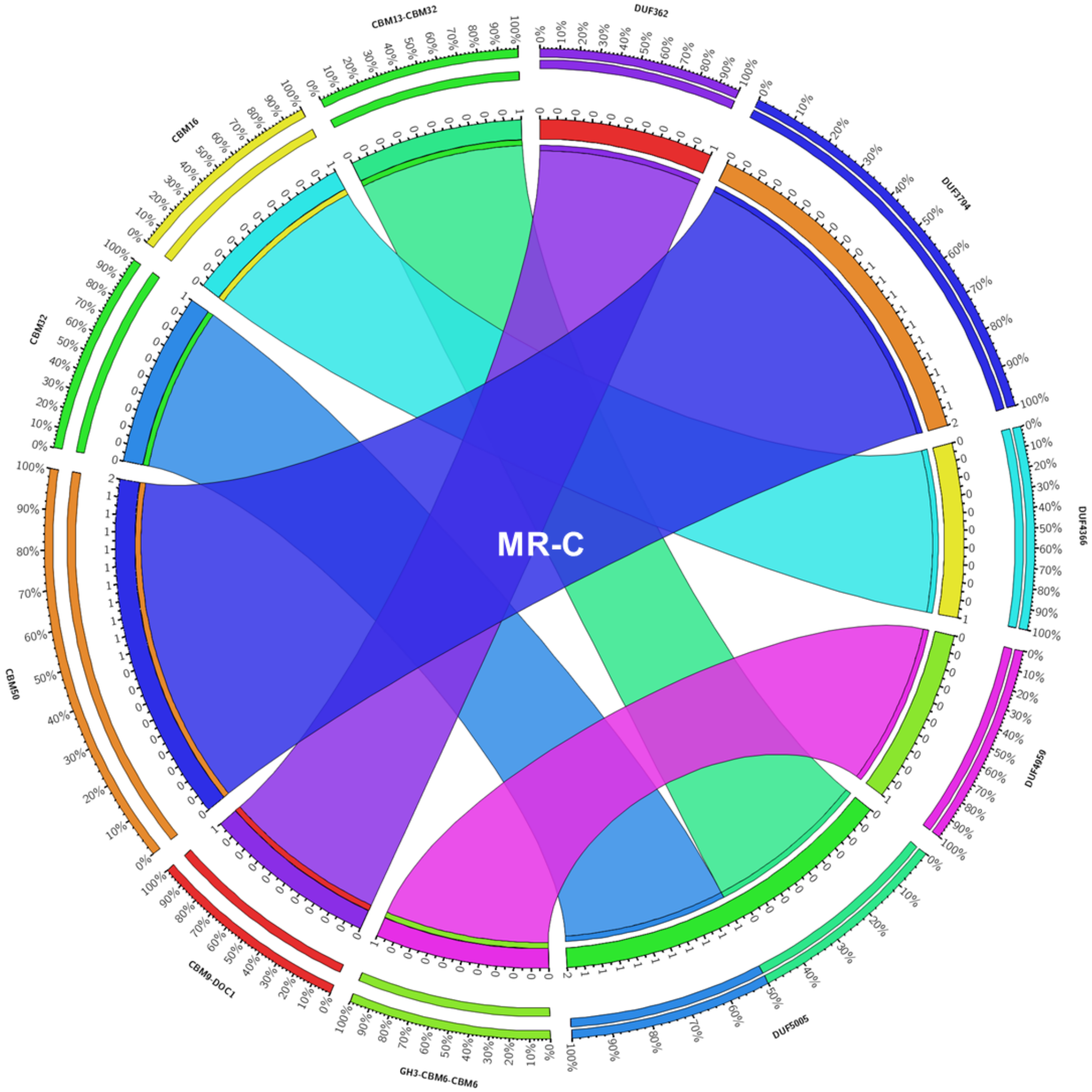
MR-C
DUF362
DUF3704
DUF4366
DUF4059
DUF5005
GH3-CBM6-CBM6
CBM9-DOC1
CBM50
CBM32
CBM16
CBM13-CBM32

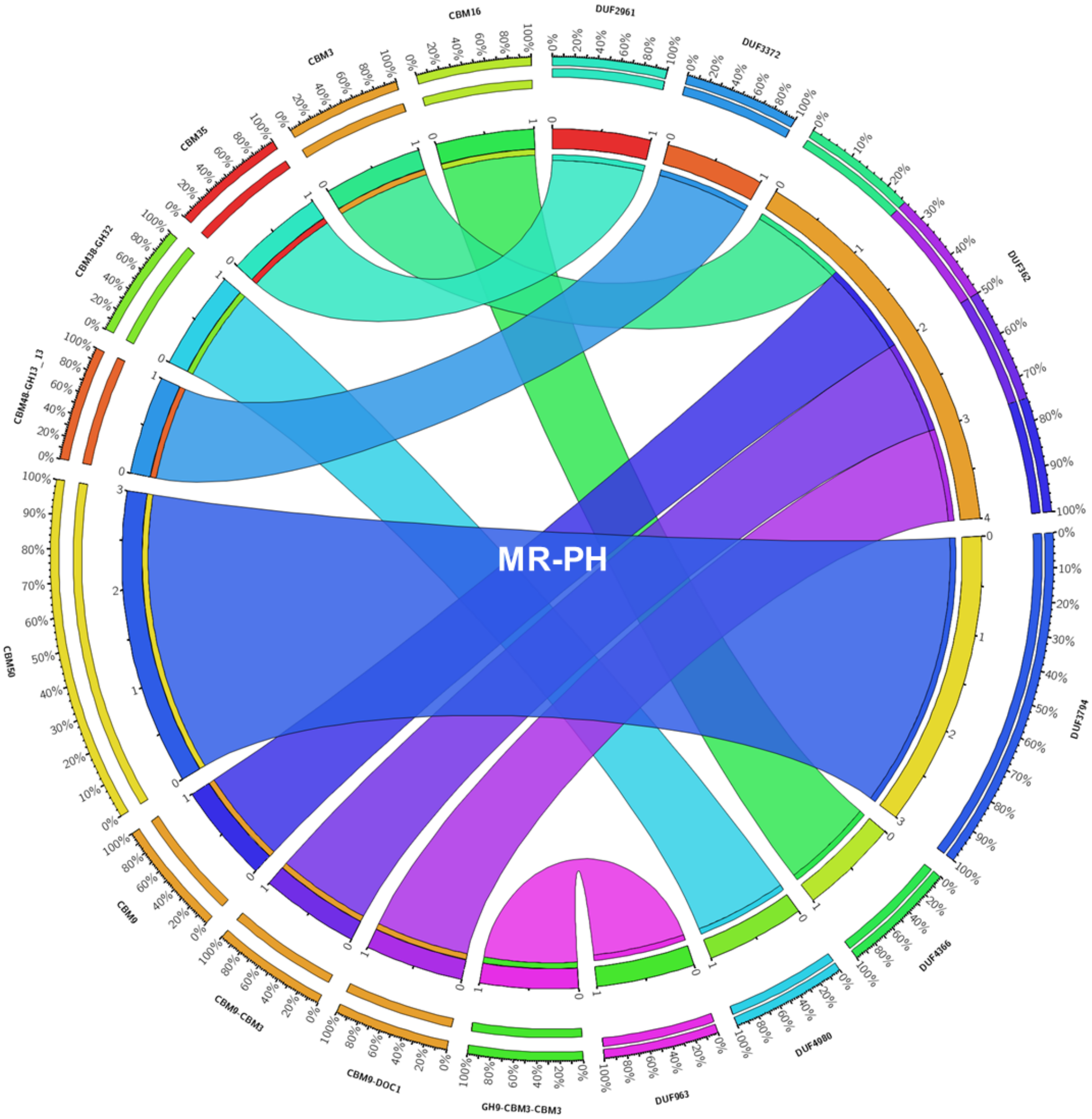

Figure S6 Carbohydrate-active proteins with domains of unknown functions identified in each metagenome

Table S1 Metagenomic DNA extraction for enrichment cultures from beaver dropping and moose rumen.

Sample	Concentration (ng/µL)	A_{260}/A_{280}
Beaver dropping-cellulose	73.7	2.00
Beaver dropping-poplar hydrolysate	47.7	2.00
Moose rumen-cellulose	43.8	1.95
Moose rumen-poplar hydrolysate	64.9	1.86

Table S2 Annotation of CAZymes and CBMs in the metagenomes.

	BD-C	BD-PH	MR-C	MR-PH
CBM2	1	6	2	6
CBM3	33	32	31	141
CBM4	17	12	10	6
CBM6	15	24	21	22
CBM8	0	0	1	2
CBM9	24	8	8	18
CBM13	4	6	8	5
CBM14	1	0	0	0
CBM16	12	4	2	6
CBM17	0	0	2	0
CBM20	3	18	5	6
CBM22	7	6	6	7
CBM23	4	0	1	0
CBM25	7	4	6	9
CBM26	0	3	0	3
CBM27	1	0	1	0
CBM28	0	0	1	0
CBM30	2	2	2	4
CBM32	23	33	15	26
CBM34	5	6	0	4
CBM35	5	10	5	12
CBM38	2	5	1	2
CBM40	0	0	0	1
CBM41	3	1	0	2

CBM44	1	1	1	1
CBM45	1	0	0	0
CBM47	1	0	0	3
CBM48	32	42	18	28
CBM50	162	179	114	173
CBM51	14	0	0	1
CBM54	0	0	4	0
CBM57	13	0	0	1
CBM58	0	3	0	4
CBM61	0	4	0	4
CBM62	0	4	2	2
CBM63	1	1	1	1
CBM66	32	11	6	5
CBM67	13	3	3	3
CBM70	1	0	0	1
CBM77	0	1	1	0
CE1	28	63	13	51
CE2	5	4	1	5
CE3	8	6	4	9
CE4	31	41	20	44
CE6	7	6	2	6
CE7	16	10	6	9
CE8	7	7	5	1
CE9	23	19	12	11
CE11	9	14	5	11
CE12	3	5	4	2
CE14	46	25	24	30
CE15	8	15	1	9
CE16	0	4	0	0
GT1	21	18	15	14
GT2	432	336	308	326
GT3	1	8	2	2
GT4	344	307	298	263
GT5	26	19	15	16
GT6	1	0	0	1
GT7	0	0	0	1
GT8	3	2	5	3
GT9	84	24	41	22
GT10	0	0	1	3
GT11	7	3	2	4
GT17	1	0	0	0
GT19	12	19	6	13
GT20	3	10	9	9

GT21	4	0	1	0
GT25	2	2	5	0
GT26	20	8	10	8
GT28	39	29	26	36
GT30	13	14	7	13
GT32	4	1	4	1
GT35	25	36	17	19
GT39	3	7	3	4
GT41	59	1	0	0
GT48	2	0	0	0
GT50	0	1	0	1
GT51	33	54	26	41
GT55	0	1	0	0
GT56	0	0	0	1
GT66	1	2	12	3
GT73	1	0	1	0
GT74	2	0	2	0
GT76	8	2	4	5
GT81	6	6	6	7
GT83	28	22	14	22
GT84	2	7	3	4
GT87	3	0	0	0
GT89	8	0	5	1
PL1	19	4	5	2
PL2	2	0	0	0
PL3	1	0	0	0
PL6	1	0	2	0
PL7	2	0	0	0
PL8	0	1	0	0
PL9	26	13	4	4
PL10	2	1	0	0
PL11	9	2	1	3
PL12	15	2	4	7
PL14	0	0	0	1
PL15	3	0	0	1
PL17	4	1	1	2
PL21	2	0	0	0
PL22	2	0	0	3
GH1	28	7	20	21
GH2	50	88	35	55
GH3	63	102	36	90
GH4	24	11	25	15
GH5	14	5	2	11

GH5_1	4	5	4	13
GH5_2	2	5	2	4
GH5_4	0	10	1	12
GH5_5	0	7	0	0
GH5_7	6	6	1	7
GH5_8	0	0	0	1
GH5_12	2	3	4	1
GH5_13	1	5	1	3
GH5_17	0	0	0	1
GH5_19	1	0	1	0
GH5_22	1	3	6	3
GH5_25	3	2	3	1
GH5_36	1	3	1	1
GH5_37	0	2	1	3
GH5_40	0	1	0	1
GH5_44	0	1	1	0
GH5_45	0	1	0	0
GH5_46	2	4	2	6
GH5_52	3	0	2	0
GH8	5	5	2	13
GH9	28	36	25	64
GH10	19	20	15	13
GH11	2	4	4	4
GH12	0	2	0	3
GH13	36	39	20	36
GH13_2	1	0	0	0
GH13_3	0	4	0	0
GH13_4	4	1	2	0
GH13_6	0	0	0	1
GH13_7	0	0	0	1
GH13_8	1	4	2	2
GH13_9	13	7	6	5
GH13_10	1	5	0	0
GH13_11	6	8	5	2
GH13_13	0	0	0	1
GH13_14	4	5	1	4
GH13_16	6	8	3	2
GH13_18	6	6	4	2
GH13_19	0	2	0	1
GH13_20	12	8	7	11
GH13_21	1	1	0	1
GH13_23	6	11	5	9
GH13_26	1	5	0	0

GH13_29	1	0	0	0
GH13_31	5	3	1	2
GH13_33	0	4	0	0
GH13_36	2	3	1	4
GH13_38	1	5	2	1
GH13_39	0	1	0	1
GH13_41	1	0	0	0
GH14	1	0	0	0
GH15	8	7	9	6
GH16	11	22	6	11
GH17	2	2	1	2
GH18	21	27	19	38
GH19	0	2	0	1
GH20	35	18	13	17
GH23	57	57	31	42
GH24	2	11	2	3
GH25	19	9	5	5
GH26	8	11	8	9
GH27	9	24	3	18
GH28	13	20	9	4
GH29	25	27	5	5
GH30	3	2	1	9
GH30_1	3	2	4	3
GH30_2	1	1	1	0
GH30_3	4	9	2	1
GH30_8	1	1	2	2
GH31	17	31	19	34
GH32	10	10	4	6
GH33	23	14	1	2
GH35	12	17	3	12
GH36	21	18	8	15
GH37	1	1	0	1
GH38	23	17	5	20
GH39	23	8	7	9
GH42	12	5	4	8
GH43	0	68	1	1
GH43_1	1	0	2	2
GH43_2	0	0	0	4
GH43_3	1	0	3	0
GH43_4	1	0	0	1
GH43_5	1	0	0	1
GH43_8	1	0	0	0
GH43_10	2	0	3	2

GH43_11	3	0	0	4
GH43_12	7	0	3	3
GH43_16	1	0	1	1
GH43_17	1	0	6	5
GH43_18	1	0	0	0
GH43_20	1	0	1	1
GH43_22	2	0	0	0
GH43_24	1	0	1	3
GH43_26	2	0	1	4
GH43_28	1	0	0	10
GH43_29	1	0	1	1
GH43_30	1	0	0	0
GH43_31	2	0	2	1
GH43_33	1	0	0	0
GH43_34	2	0	0	0
GH43_35	2	0	1	4
GH44	2	2	2	3
GH46	0	0	0	1
GH48	2	2	6	18
GH50	4	4	3	6
GH51	20	28	12	20
GH52	2	2	5	2
GH53	3	20	3	21
GH55	2	4	0	1
GH57	41	19	19	18
GH62	1	1	1	1
GH63	14	7	8	4
GH65	5	12	9	17
GH66	2	1	3	1
GH67	6	10	3	3
GH73	18	12	2	10
GH74	33	3	20	7
GH76	3	5	4	2
GH77	15	25	14	15
GH78	21	32	3	4
GH81	3	1	1	1
GH84	5	1	0	0
GH87	0	2	1	1
GH88	19	6	1	2
GH89	1	1	1	0
GH92	18	43	9	9
GH93	0	4	1	0
GH94	24	22	18	26

GH95	8	17	3	4
GH96	0	0	0	1
GH97	10	14	2	4
GH98	0	1	0	0
GH99	2	1	0	1
GH100	1	0	0	0
GH102	0	1	0	3
GH103	1	9	0	1
GH105	9	18	12	8
GH106	4	23	0	0
GH107	1	0	0	0
GH108	6	1	1	0
GH109	7	8	1	5
GH110	3	1	0	0
GH112	1	0	0	0
GH113	5	1	4	3
GH114	1	0	0	0
GH115	5	9	3	4
GH116	7	7	4	4
GH117	2	0	0	0
GH119	1	0	0	0
GH120	2	0	1	1
GH121	0	0	1	0
GH123	11	4	0	3
GH125	4	5	5	4
GH126	0	0	1	0
GH127	14	27	2	21
GH128	0	2	2	0
GH129	6	0	0	1
GH130	26	27	19	21
GH133	4	12	5	3
GH139	1	0	0	0
GH140	0	2	0	0
GH141	1	2	1	1
GH142	1	1	0	0

Table S3 Relative abundances of polysaccharide-active CAZymes that contributed to >0.2 or <-0.2 component loading in PC1, PC2, and PC3 in the PCA plot.

	Component loading[1]			Relative abundances of plant polysaccharide-active CAZys annotated in metagenomes (%)										
	PC1	PC2	PC3	BD-C	MR-C	BD-PH	MR-PH	Moose	Cow	Reindeer	Wallaby	Sheep	Panda	Termite
GH3	0.36	-0.49	-0.25	2.4	2.2	4.0	4.3	3.5	4.7	5.5	4.9	3.5	1.5	5.1
GH5	0.31			1.5	2.0	2.5	3.2	2.2	2.7	2.2	1.7	1.6	0.3	6.2
GH2	0.29	0.31		1.9	2.1	3.4	2.6	3.9	4.3	4.2	4.0	5.2	1.1	2.8
GH43	0.29	0.36	-0.47	1.4	1.6	2.7	2.3	5.2	4.5	6.1	4.0	2.9	2.4	3.3
CE1			0.22	1.1	0.8	2.5	2.4	1.0	1.9	1.6	1.3	1.5	0.1	0.3
GH9		-0.28		1.1	1.5	1.4	3.0	0.5	1.6	0.9	0.4	0.6	0.0	2.1
GH94		-0.38	-0.27	0.9	1.1	0.9	1.2	0.6	1.1	1.2	1.3	0.8	0.9	4.3
GH10		-0.33	-0.23	0.7	0.9	0.8	0.6	0.8	1.6	1.0	0.8	0.8	0.1	4.1
GH78		0.26		0.8	0.2	1.3	0.2	1.3	1.6	1.2	2.4	2.1	0.3	0.2
CE4	-0.31			1.2	1.2	1.6	2.1	0.6	0.9	0.4	1.0	1.6	4.4	0.9
GH1	-0.58		-0.61	1.1	1.2	0.3	1.0	0.8	0.4	0.6	3.4	0.7	7.4	1.2

[1]Only values >0.2 or <-0.2 are shown.

Table S4 Domain architecture, number of gene count and relative abundances of top 20 most abundant multi-modular CAZymes in the metagenomes.

	BD-C		BD-PH		MR-C		MR-PH	
**Multimodular CAZymes**	**Count**	**%**	**Count**	**%**	**Count**	**%**	**Count**	**%**
CBM50-CBM50-GH18	2	1.2%	12	6.2%	5	5.8%	11	5.0%
CBM48-GH13_9	13	7.6%	7	3.6%	6	7.0%	4	1.8%
CBM48-GH13_11	6	3.5%	7	3.6%	5	5.8%	2	0.9%
CBM48-CE1	1	0.6%	8	4.1%	0	0.0%	10	4.6%
CBM67-GH78	9	5.3%	2	1.0%	3	3.5%	3	1.4%
GT4-GT4	4	2.3%	4	2.1%	1	1.2%	7	3.2%
GT2-GT2	7	4.1%	0	0.0%	5	5.8%	3	1.4%
GH9-CBM3-CBM3	0	0.0%	0	0.0%	0	0.0%	14	6.4%
GH9-CBM3	2	1.2%	2	1.0%	2	2.3%	7	3.2%
GT84-GH94	1	0.6%	6	3.1%	2	2.3%	3	1.4%
GH23-CBM50-CBM50	2	1.2%	4	2.1%	3	3.5%	3	1.4%
CBM48-GH13	3	1.8%	2	1.0%	4	4.7%	2	0.9%
GH43_28-CBM32	0	0.0%	5	2.6%	0	0.0%	6	2.8%
CBM34-GH13_20	3	1.8%	4	2.1%	0	0.0%	3	1.4%
CBM66-PL9	10	5.8%	0	0.0%	0	0.0%	0	0.0%
CBM66-PL1	10	5.8%	0	0.0%	0	0.0%	0	0.0%
CBM20-CBM20-GH77	1	0.6%	7	3.6%	2	2.3%	0	0.0%
GT2-GT4	5	2.9%	1	0.5%	3	3.5%	0	0.0%
GH9-CE4	1	0.6%	2	1.0%	1	1.2%	5	2.3%
CBM50-GH18	3	1.8%	1	0.5%	1	1.2%	3	1.4%

Table S5 Domain architecture for all CAZy-dockerins identified in the metagenomes.

Domain architecture	BD-C	BD-PH	MR-C	MR-PH
CBM13-DOC1	1	1	1	1
CBM30-GH9-DOC1	2	2	2	2
CBM35-DOC1	1	1	1	1
CBM35-GH26-DOC1	1	1	1	1
CBM4-DOC1	0	0	2	0
CBM4-GH9-DOC1	1	1	1	1
CBM6-DOC1	0	0	1	0
CBM9-DOC1	1	1	1	4
CE12-DOC1	2	2	2	2
CE15-DOC1	1	1	1	1
CE1-DOC1	2	2	2	2
CE3-DOC1	3	3	2	3
CE8-DOC1	1	1	1	1
DOC1-CBM4-GH16	1	1	1	1
DOC1-CBM63	1	1	1	1
DOC1-CE2	1	1	1	1
DOC1-CE6	1	1	1	1
DOC1-GH3	0	0	0	1
DOC1-GH3-CBM4-CBM4-CBM4-GH9	1	1	1	1
GH10-DOC1-CE3	1	1	1	1
GH11-CBM6-DOC1-CE6	1	1	1	1
GH11-DOC1-GH10	1	1	1	1
GH16-DOC1	1	1	1	1
GH18-DOC1	1	1	1	1
GH28-DOC1	1	1	1	1
GH30_8-DOC1	1	1	1	1
GH30-DOC1	1	1	1	1
GH31-CBM32-DOC1	1	0	0	0
GH43_10-CBM6-DOC1	1	1	1	1
GH43_20-DOC1	1	1	1	1
GH43_24-CBM13-DOC1	0	0	1	0
GH43_24-DOC1	1	1	0	1
GH43_26-DOC1	1	1	1	1
GH44-DOC1	1	1	1	1
GH48-DOC1	2	2	2	2
GH5_1-DOC1	2	2	2	2
GH53-DOC1	1	1	1	1

GH5-CBM32-DOC1	1	1	1	1
GH5-DOC1	0	0	0	1
GH62-CBM6-DOC1-CE3	1	1	1	1
GH8-DOC1	1	1	1	1
GH8-DOC1-CE4	1	1	1	1
GH9-CBM3-CBM3-DOC1	3	3	3	3
GH9-CBM3-DOC1	7	7	7	7
GH9-DOC1	3	3	3	3
PL11-DOC1	2	2	1	2
PL1-DOC1-PL9	1	1	1	1

Table S6 Complete list of components identified in the predicted PULs in the metagenomes.

	BD-C	BD-PH	MR-C	MR-PH
Anti-σ	13	29	9	10
AraC	0	1	0	0
ECF-σ	14	25	10	10
GntR	0	1	1	0
GT2	1	1	0	0
HTCS	2	28	2	7
MFS	2	18	5	10
Pept_CA	0	2	0	0
Pept_MC	0	1	2	2
Pept_MH	0	2	0	0
Pept_na	0	0	2	2
Pept_PB	0	1	1	1
Pept_PC	0	0	1	1
Pept_SC	1	7	0	8
Pept_SE	0	2	0	0
Sulf_1	0	5	1	0
Sulf_4	0	1	0	4
SusC	35	196	37	67
SusD	35	187	35	65
SusR	0	12	3	4
CBM4	0	3	1	0
CBM6	0	9	5	3
CBM13	0	1	1	0
CBM20	0	0	1	1
CBM32	0	9	2	3
CBM35	0	3	1	0
CBM38	0	4	1	2
CBM48	1	9	0	7
CBM58	0	3	0	4
CBM62	0	1	1	0
CBM66	0	3	3	0
CBM67	0	1	2	0
CBM77	0	1	1	0
CE1	2	28	0	24
CE4	0	2	0	1

CE6	0	3	0	3
CE7	1	1	0	0
CE8	0	5	4	0
CE12	0	2	2	0
CE15	0	1	0	3
GH2	2	34	5	3
GH3	5	23	6	8
GH5	0	8	5	0
GH9	0	6	0	3
GH10	0	8	2	0
GH11	0	1	1	0
GH13	1	13	0	14
GH16	1	10	3	4
GH20	0	3	2	0
GH26	1	4	2	0
GH27	0	5	2	1
GH28	0	4	2	0
GH29	0	5	1	0
GH30_3	2	3	2	0
GH31	0	9	6	4
GH32	1	2	1	1
GH33	0	4	0	0
GH35	0	7	1	1
GH36	0	6	0	1
GH38	0	1	2	0
GH42	0	1	0	0
GH43	1	40	7	13
GH50	0	1	0	1
GH51	0	13	1	4
GH53	0	9	0	2
GH55	0	3	0	0
GH63	1	0	0	0
GH66	0	1	1	0
GH67	0	2	1	1
GH76	2	2	2	0
GH77	0	0	1	1
GH78	1	10	2	0
GH81	0	1	1	0
GH87	0	1	1	0
GH88	0	2	1	0
GH92	4	10	6	0

GH93	0	3	1	0
GH95	0	5	1	1
GH97	0	8	1	1
GH98	0	1	0	0
GH99	0	1	0	0
GH105	0	8	1	0
GH106	0	3	0	0
GH108	0	1	0	0
GH109	1	1	0	0
GH115	0	3	2	0
GH116	0	1	0	0
GH123	0	2	0	0
GH125	1	0	1	0
GH127	0	9	0	3
GH128	0	2	2	0
GH130	2	8	2	1
PL1	0	3	4	0
PL6	0	0	2	0
PL8	0	1	0	0
PL9	0	2	0	0
PL10	0	1	0	0
PL12	0	0	1	0
PL17	0	1	1	0
